# Supplementary material for: Brain somatic mutations observed in Alzheimer’s disease associated with aging and dysregulation of tau phosphorylation
Source: Nat Commun. 2019 Jul 12;10:3090. doi: 10.1038/s41467-019-11000-7 (PMC6626023; doi:10.1038/s41467-019-11000-7)
Supplement: Supplementary file 1 — Supplementary Information [file 41467_2019_11000_MOESM1_ESM.docx]

**Supplementary Information**

Brain somatic mutations associated with aging contribute to dysregulation of Tau phosphorylation in Alzheimer’s disease

Jun Sung Park,^1,#^ Junehawk Lee,^2,#^ Eun Sun Jung,^3,4^ Myeong-Heui Kim,^1^ Il Bin Kim,^5^ Hyeonju Son,^6^ Sangwoo Kim,^6^ Sanghyeon Kim,^7^ Young Mok Park,^8^ Inhee Mook-Jung,^3,4^ Seok Jong Yu,^2,*^ and Jeong Ho Lee^1,5,*^

1 Biomedical Science and Engineering Interdisciplinary Program, Korea Advanced Institute of Science and Technology (KAIST), Daejeon 34141, Republic of Korea

2 Center for Supercomputing Applications, Division of National Supercomputing, Korea Institute of Science and Technology Information, Daejeon 34141, Republic of Korea

3 Department of Biochemistry & Biomedical Sciences, College of Medicine, Seoul National University, Seoul 03080, Republic of Korea

4 Neuroscience Research Institute, College of Medicine, Seoul National University, Seoul, Republic of Korea

5 Graduate School of Medical Science and Engineering, Korea Advanced Institute of Science and Technology (KAIST), Daejeon 34141, Republic of Korea

6 Department of Biomedical Systems Informatics, Brain Korea 21 PLUS Project for Medical Science, Yonsei University College of Medicine, Seoul, South Korea

7 Stanley Medical Research Institute (SMRI), Laboratory of Brain Research, 9800 Medical Center Drive, Suite C-050, Rockville MD 20850, United States

8 Center for Cognition and Sociality, Institute for Basic Science (IBS), Daejeon 305-811, Republic of Korea

^#^These authors contributed equally to this work: J.S.P., J.L.

*Correspondence: [jhlee4246@kaist.ac.kr](mailto:jhlee4246@kaist.ac.kr) (J.H.L.), [seokjongyu@gmail.com](mailto:seokjongyu@gmail.com) (S.J.Y.)

**Supplementary Figures**

Supplementary Figure 1: Detailed LCM protocol for isolating genomic DNA from hippocampal formation and blood samples

Supplementary Figure 2: Targeted amplicon sequencing results to determine the EBscore threshold

Supplementary Figure 3: IGV browser images of validated brain somatic mutations

Supplementary Figure 4: Comparison of the detection accuracy and mutation profile of unmatched and matched samples

Supplementary Figure 5: Random permutation and gene-length adjusted enrichment test of genes with putatively pathogenic somatic mutations

Supplementary Figure 6: Targeted amplicon sequencing of AT8-negative/positive neurons captured with laser microdissection

Supplementary Figure 7: Protein structure-based stability prediction of wild-type and mutant PIN1 (p.Thr152Met)

Supplementary Figure 8: RT-qPCR analysis of human PIN1 constructs and knock-down efficiencies of murine Pin1 shRNAs

Supplementary Figure 9: Basal fluorescence intensities of Tau-BiFC cell lines

Supplementary Figure 10: Somatic mutation accumulation speed and clinical onset timing of Alzheimer’s disease

Supplementary Figure 11: Comparison of sub-clones and inferred clonal lineage trees in brain and blood samples of Alzheimer’s disease

Supplementary Figure 12: APP gencDNA identified from Lee et. al (Nature 2018) and our cohort


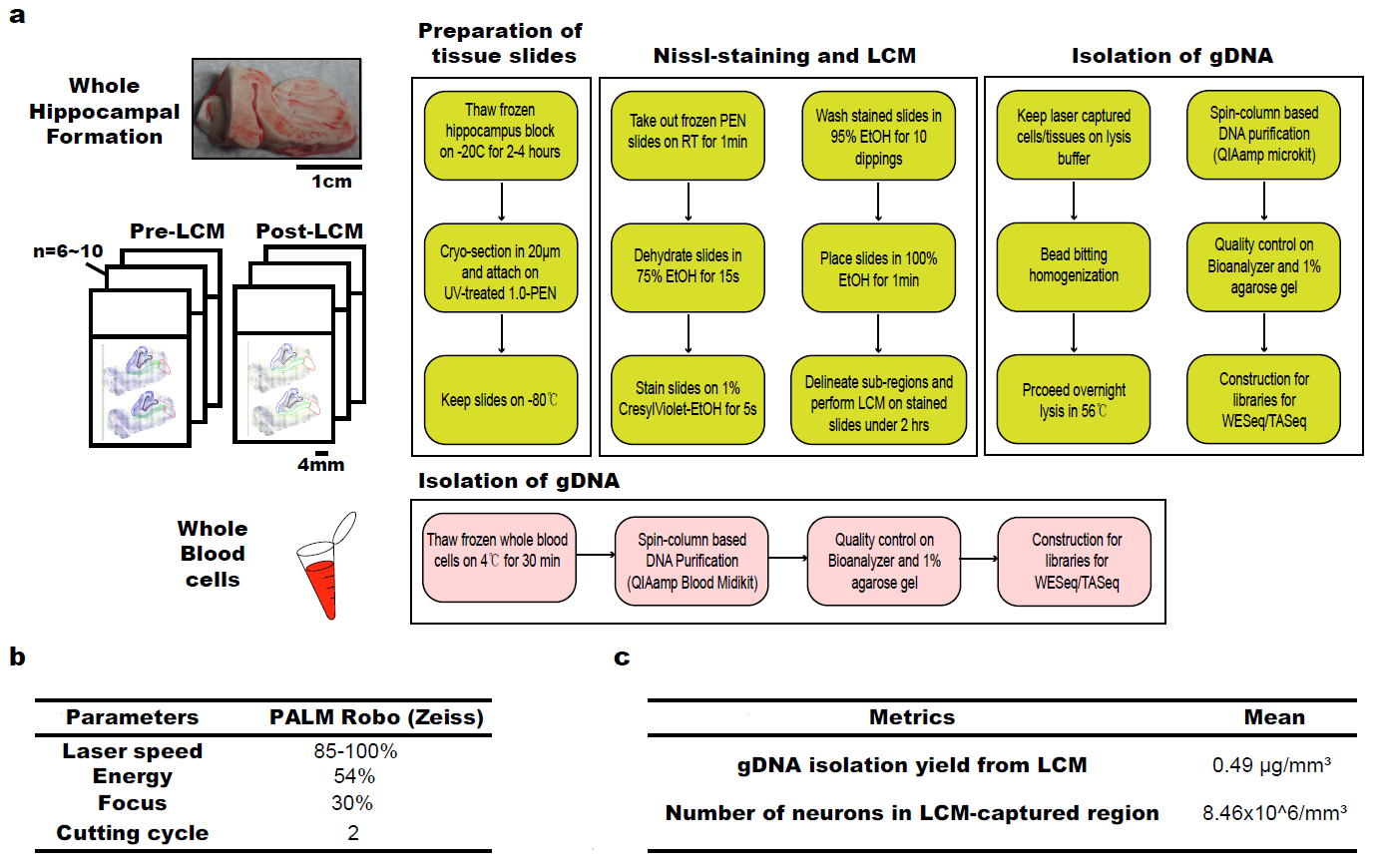


**Supplementary Figure 1: Detailed LCM protocol for isolating genomic DNA from hippocampal formation and blood samples.**

**a,** Step-by-step protocol for extracting genomic DNA from freshly frozen whole hippocampal formation tissue blocks and blood cells. **b,** Optimized laser setting parameters for microdissection on Nissl-stained tissue slides. **c,** Estimation of average gDNA yield and the number of neurons from laser capture microdissected hippocampal formation. To safely ensure 1ug of gDNA for constructing deep WES libraries, we used six to 10 stained slides, and the LCM-captured regions roughly contained ~23.5 million neurons.


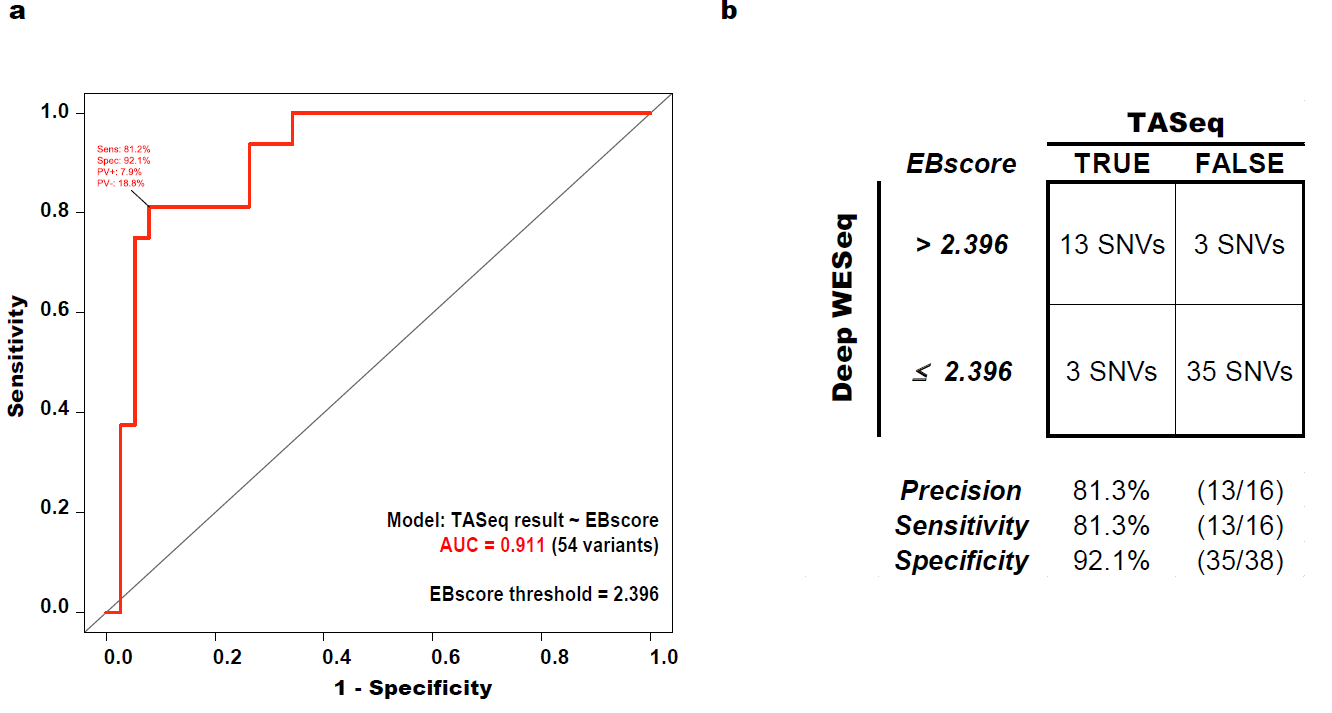


**Supplementary Figure 2: Targeted amplicon sequencing results to determine the EBscore threshold.**

**a,** A receiver operator characteristic (ROC) curve was drawn to determine the cut-off value of EBscore, which was obtained using deep whole sequencing data for 21 healthy control brains as a panel of normals. **b,** The results of targeted amplicon sequencing for detecting true calls from randomly picked 54 brain somatic mutations from 11 schizophrenia brains. The cut-off value of EBscore was set at >2.396 for maximizing the sum of sensitivity and specificity.


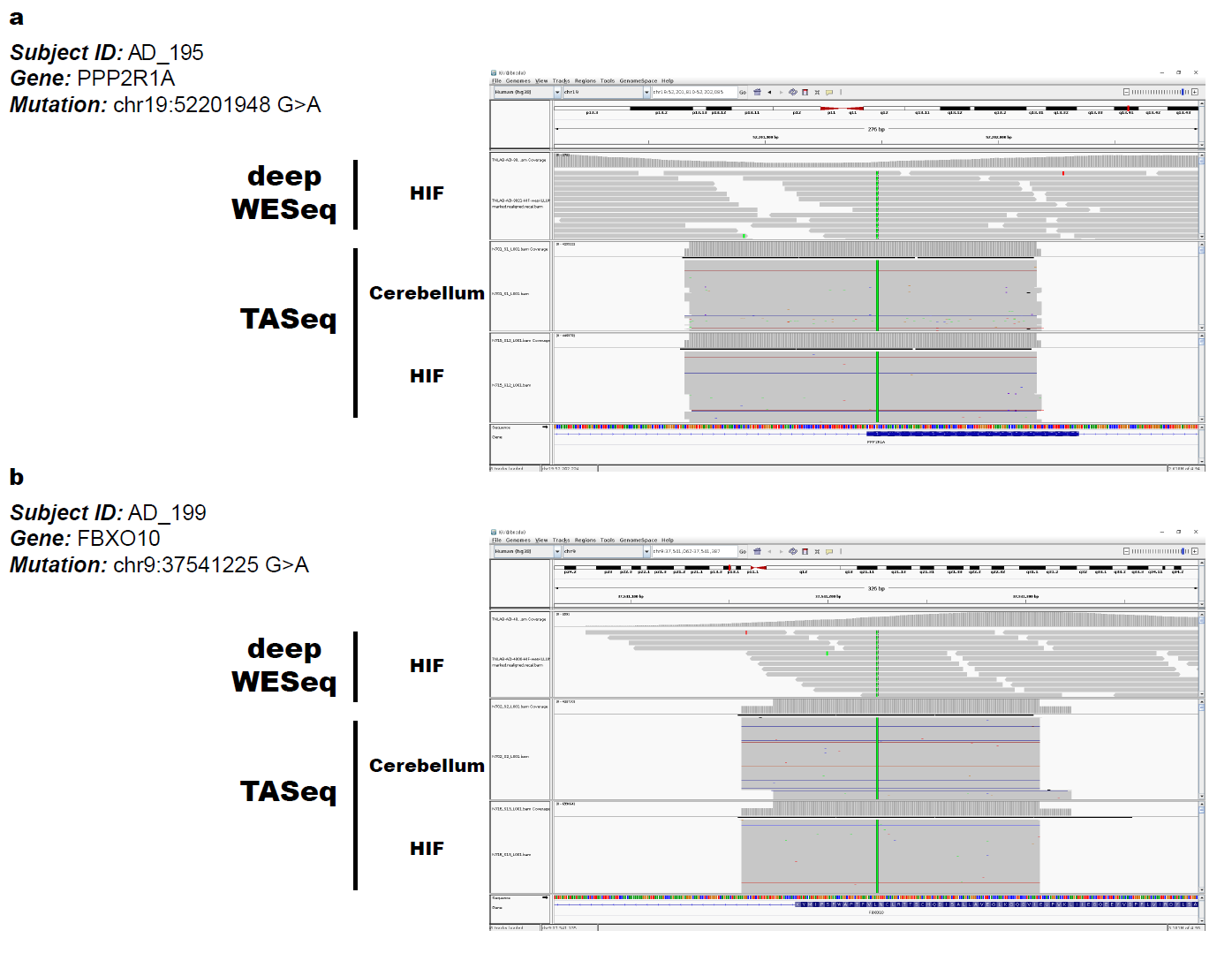


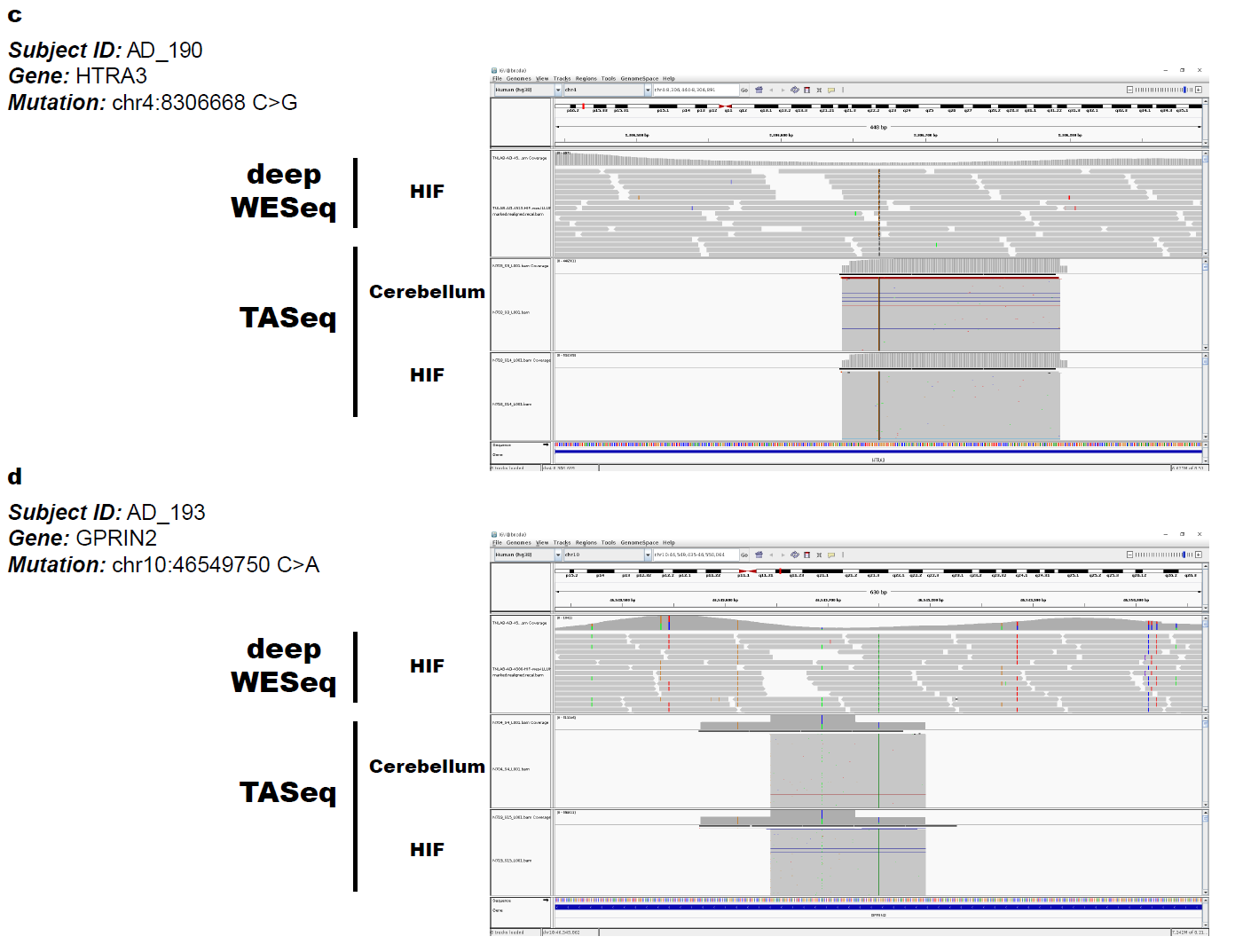


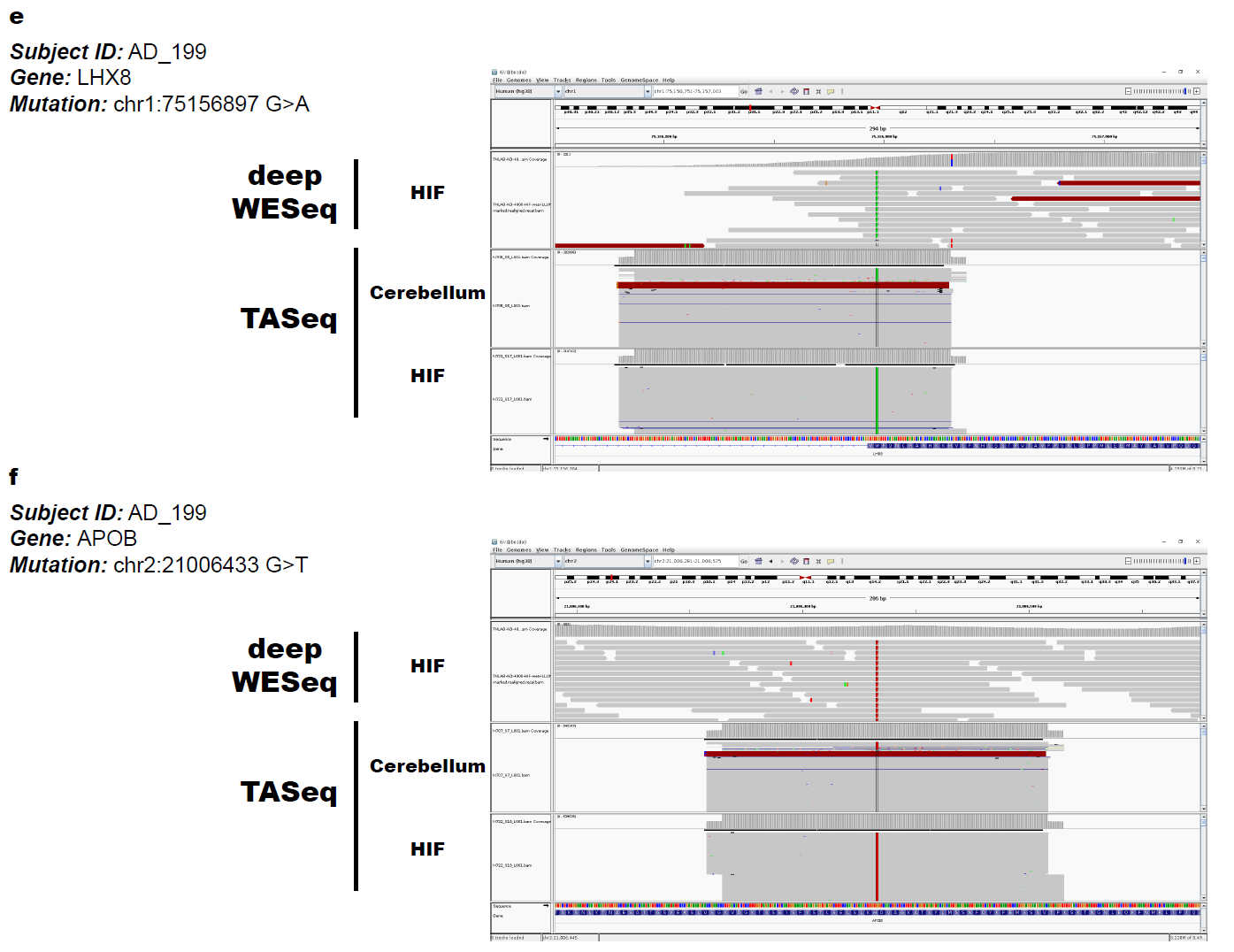


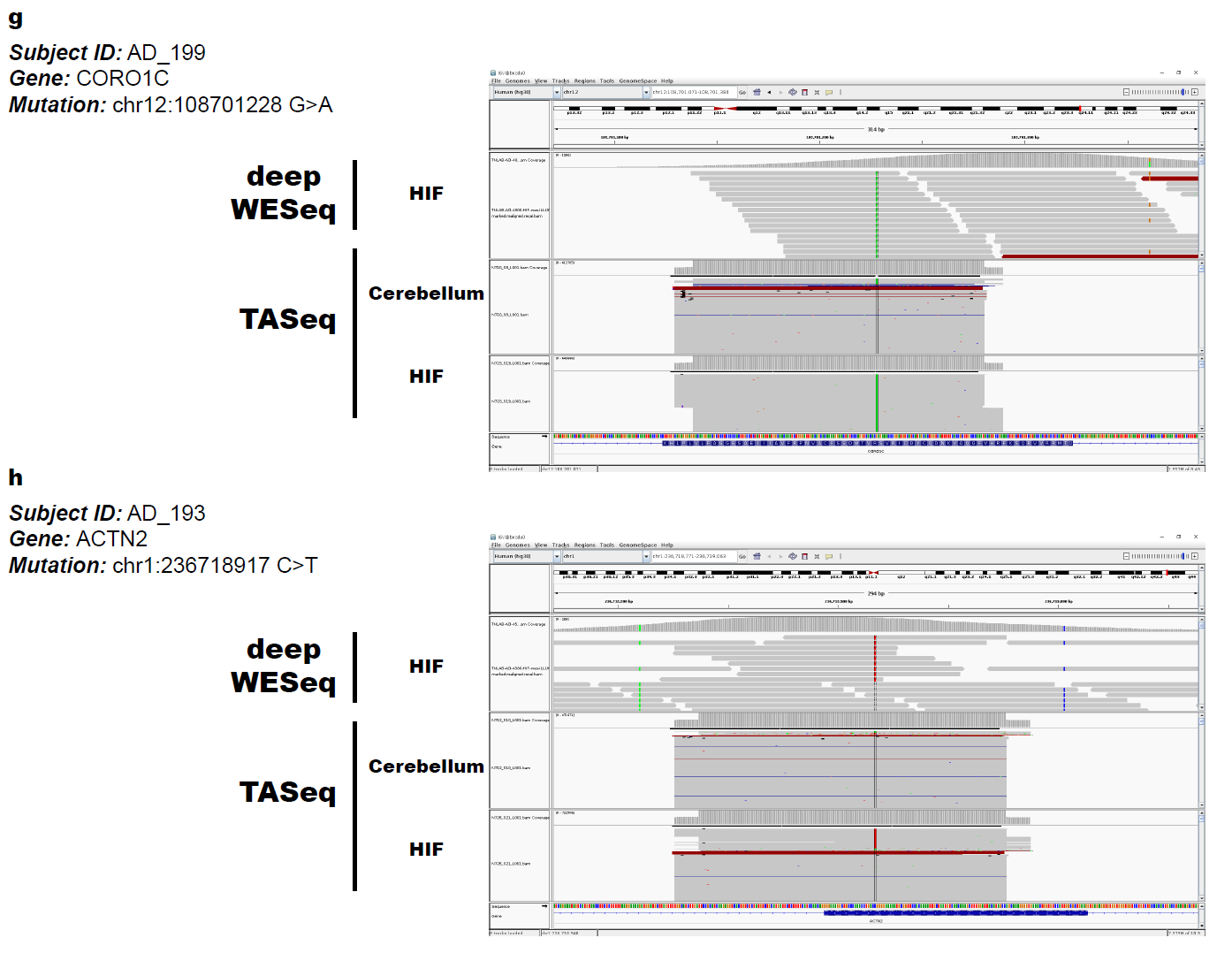


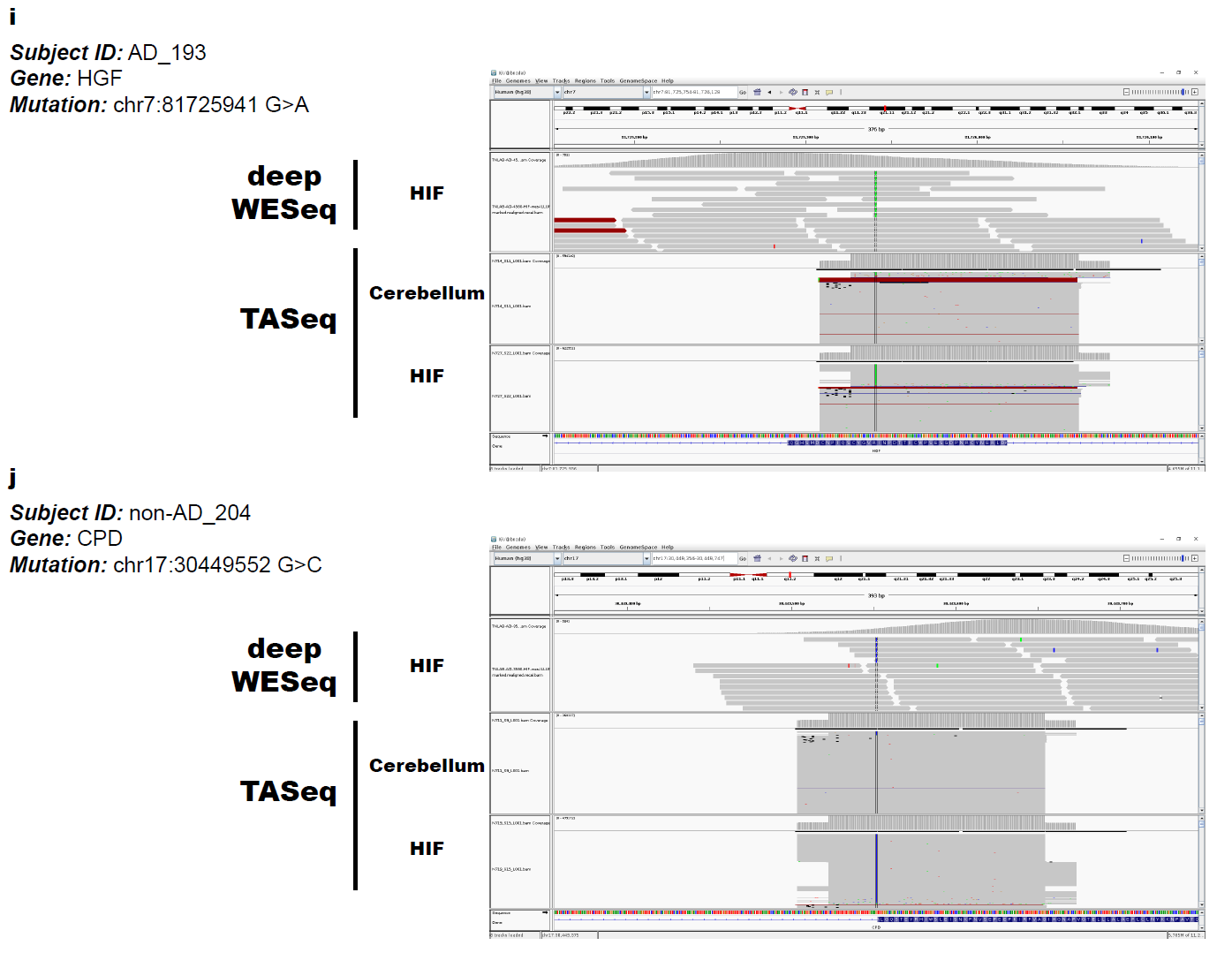


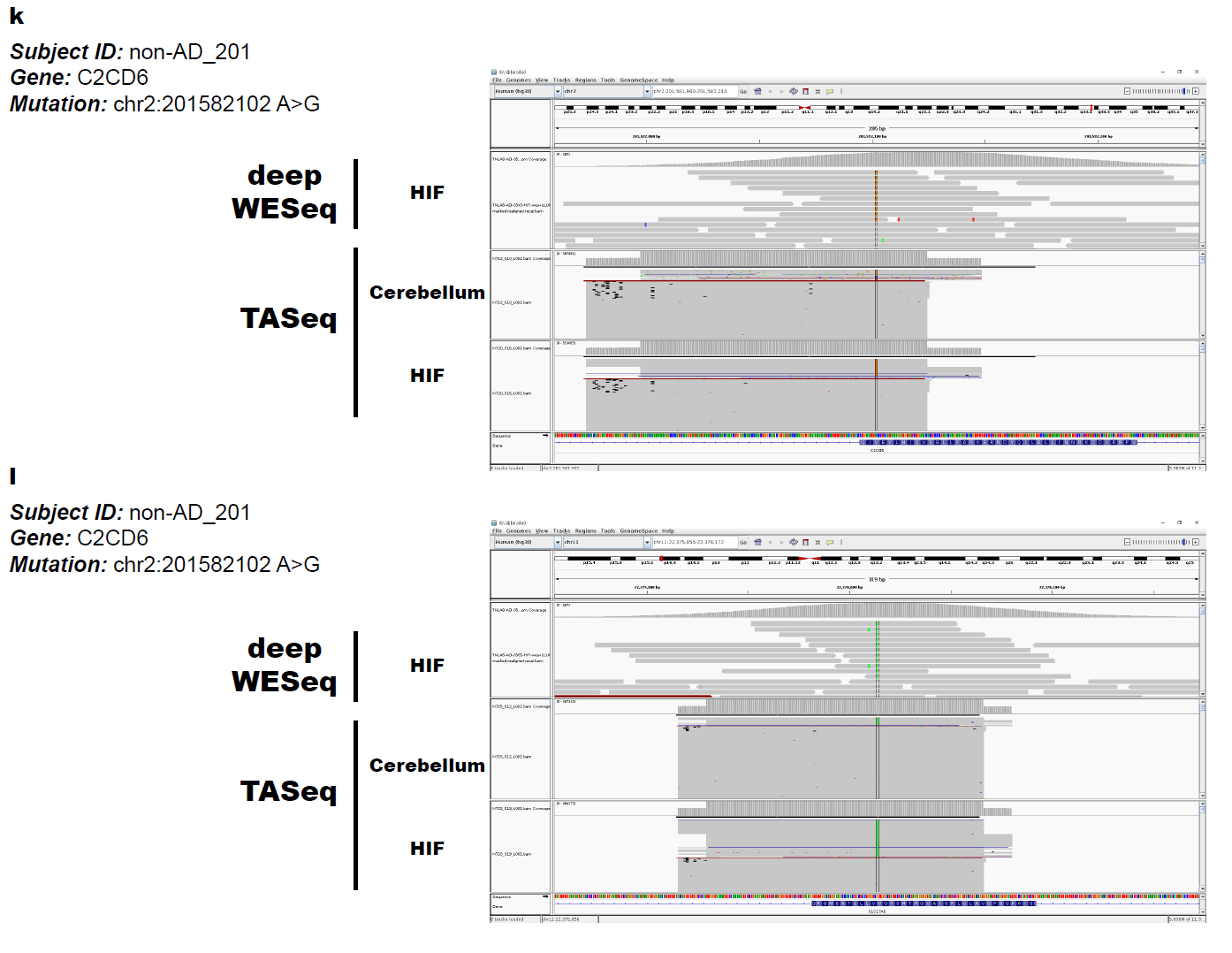


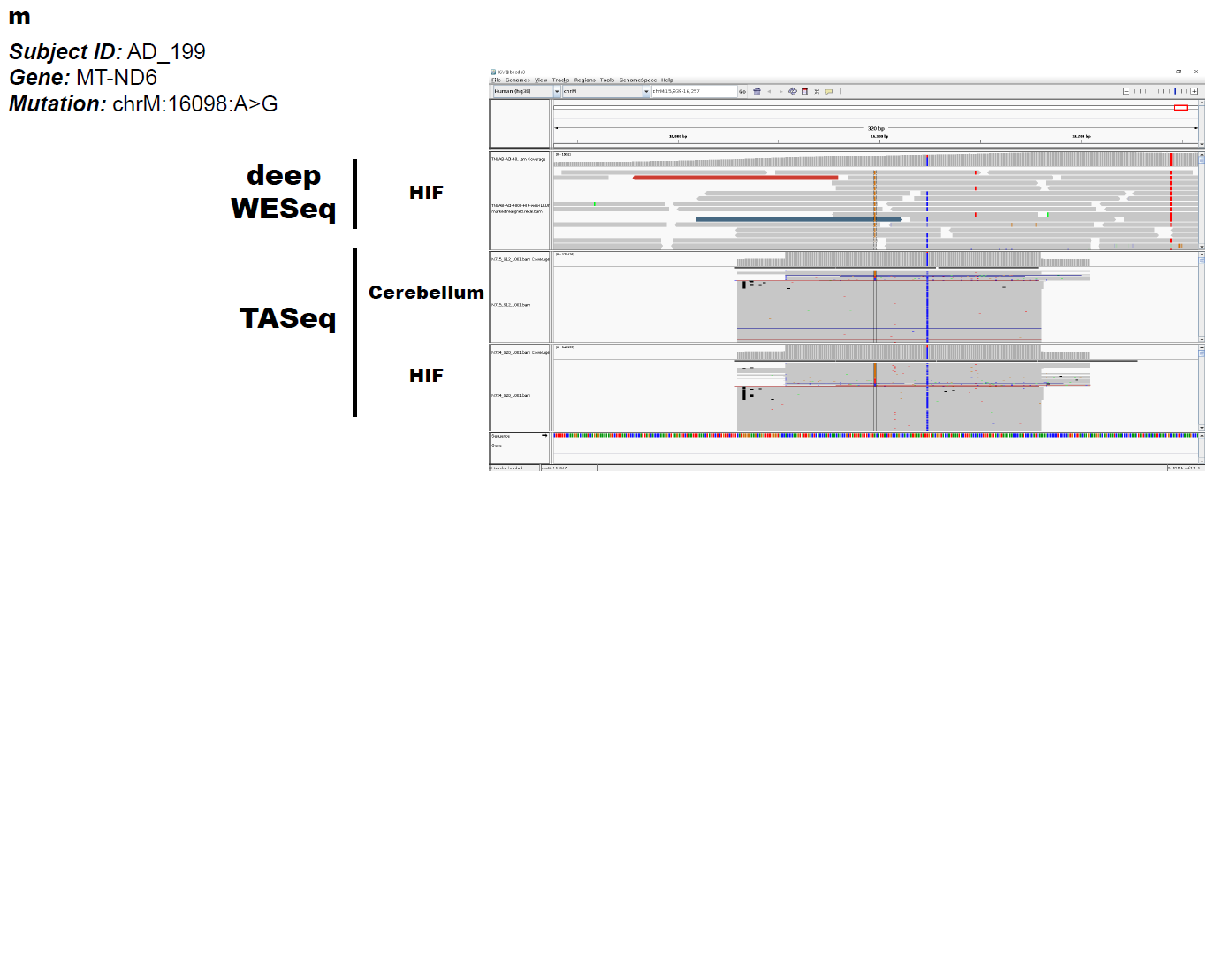


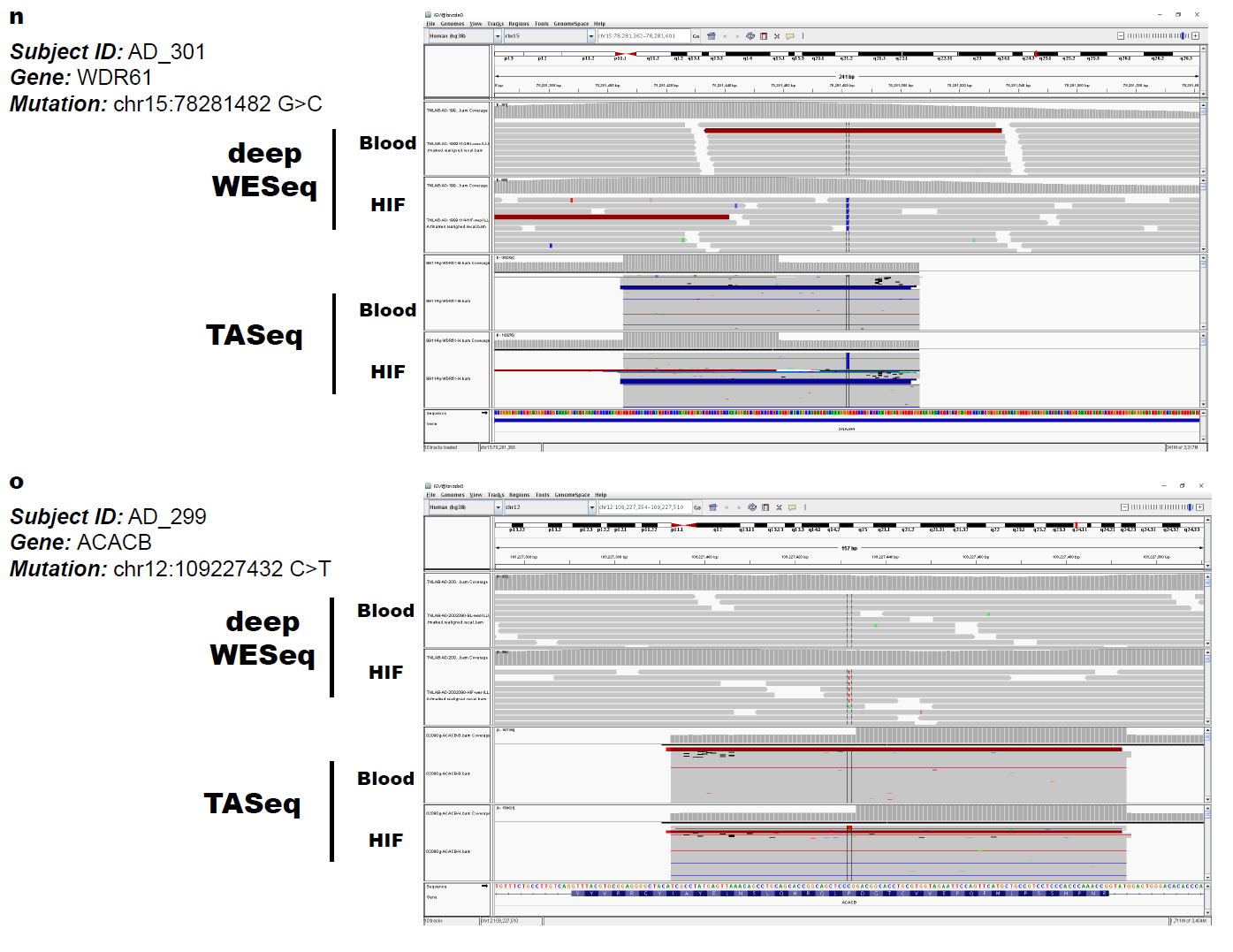


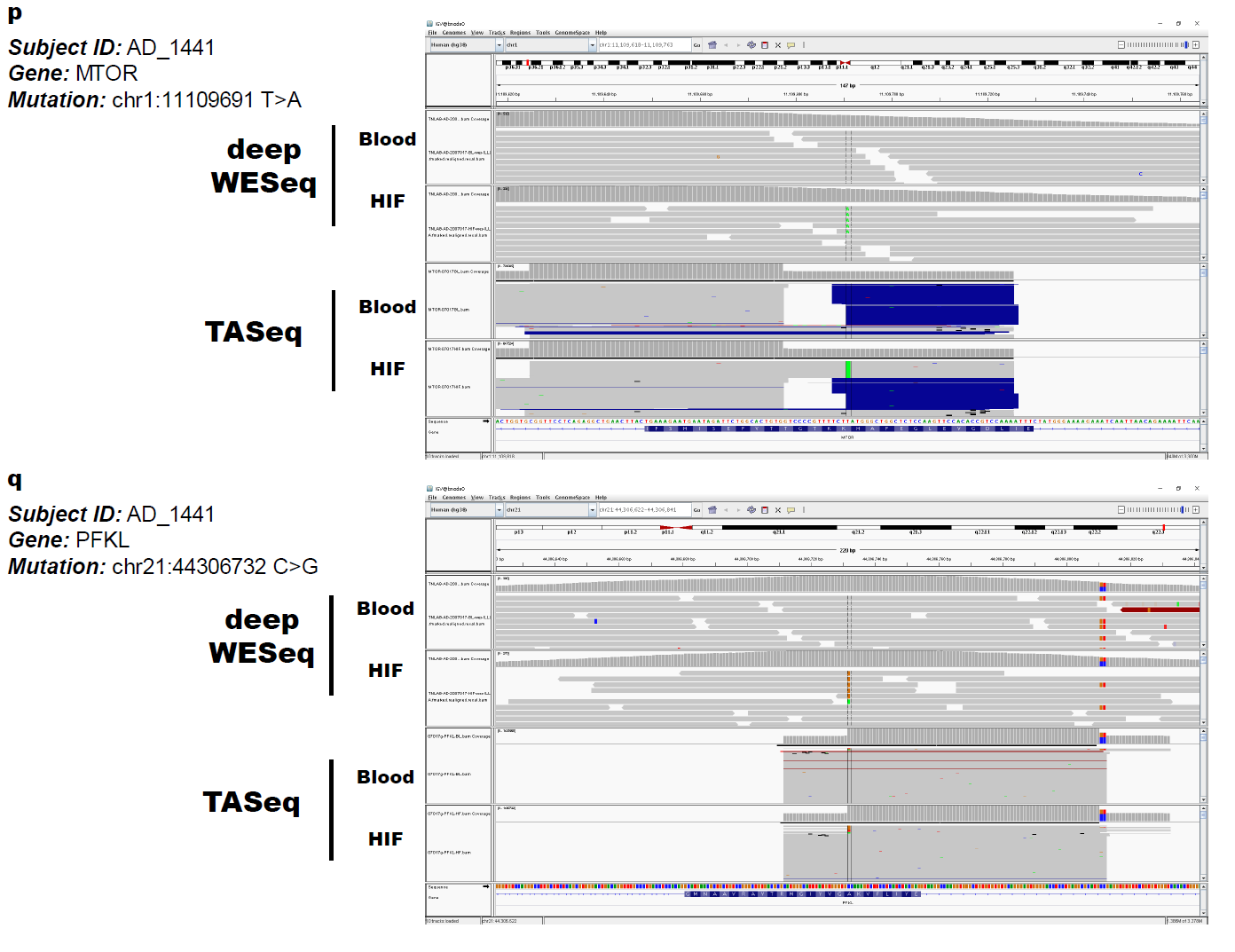


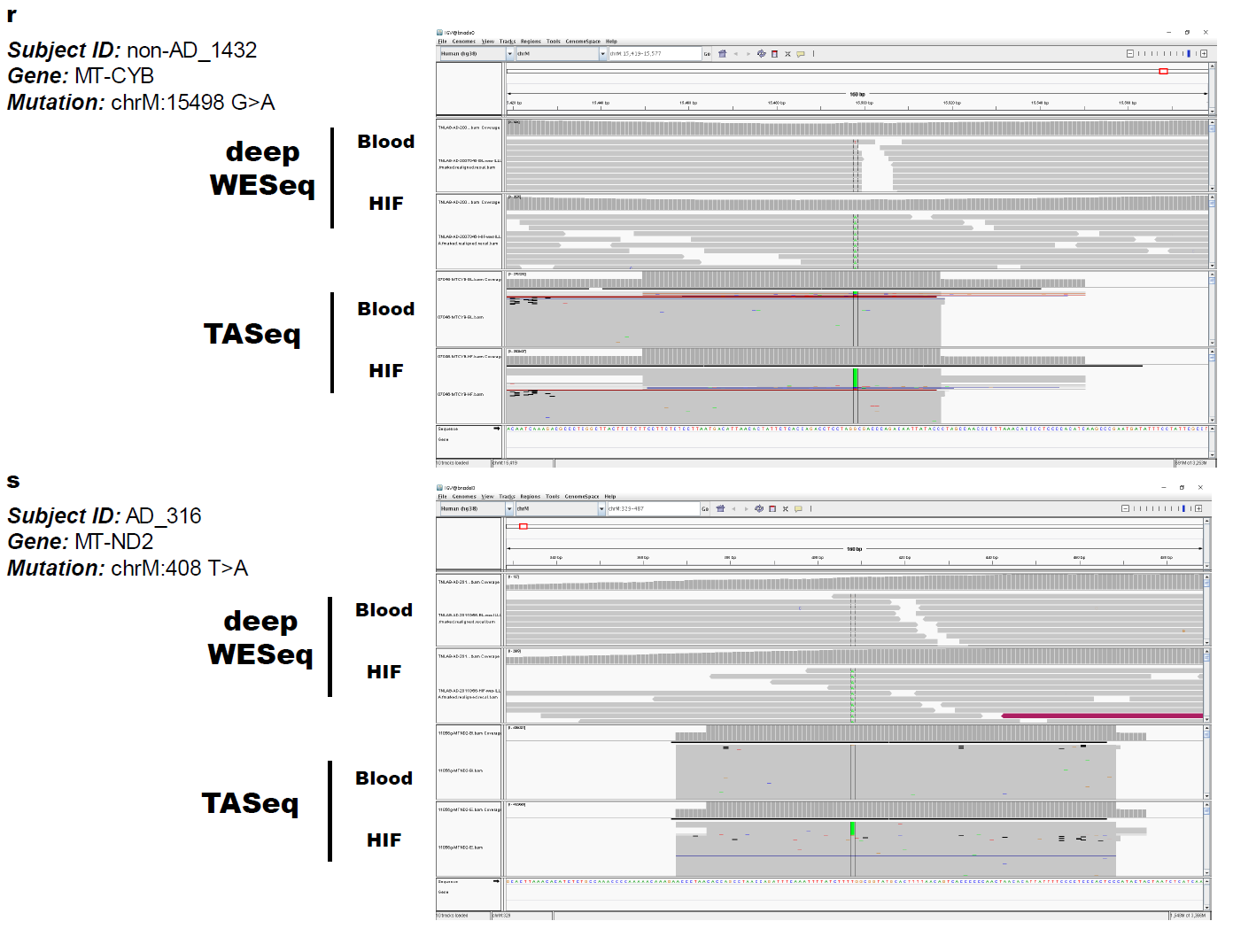


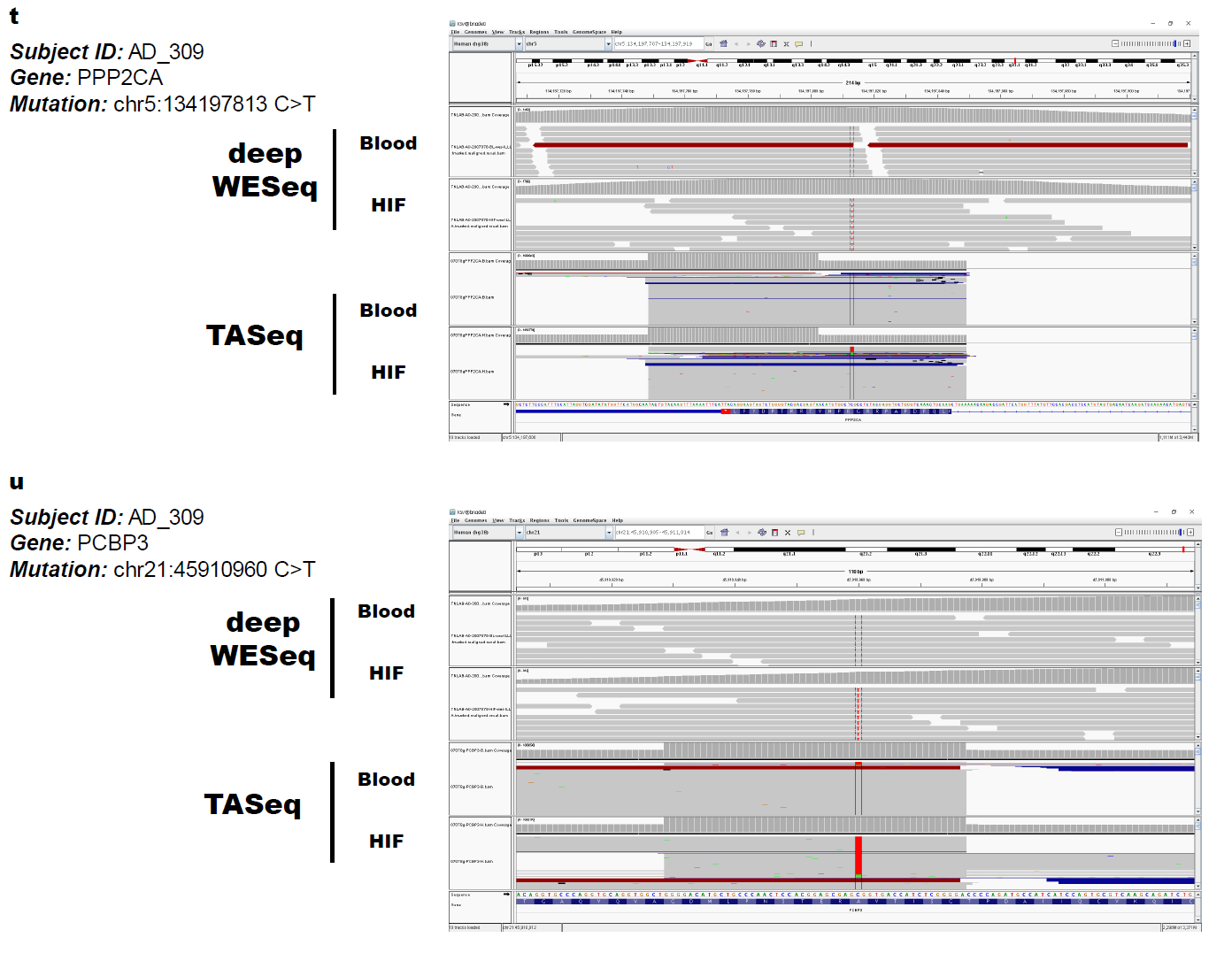


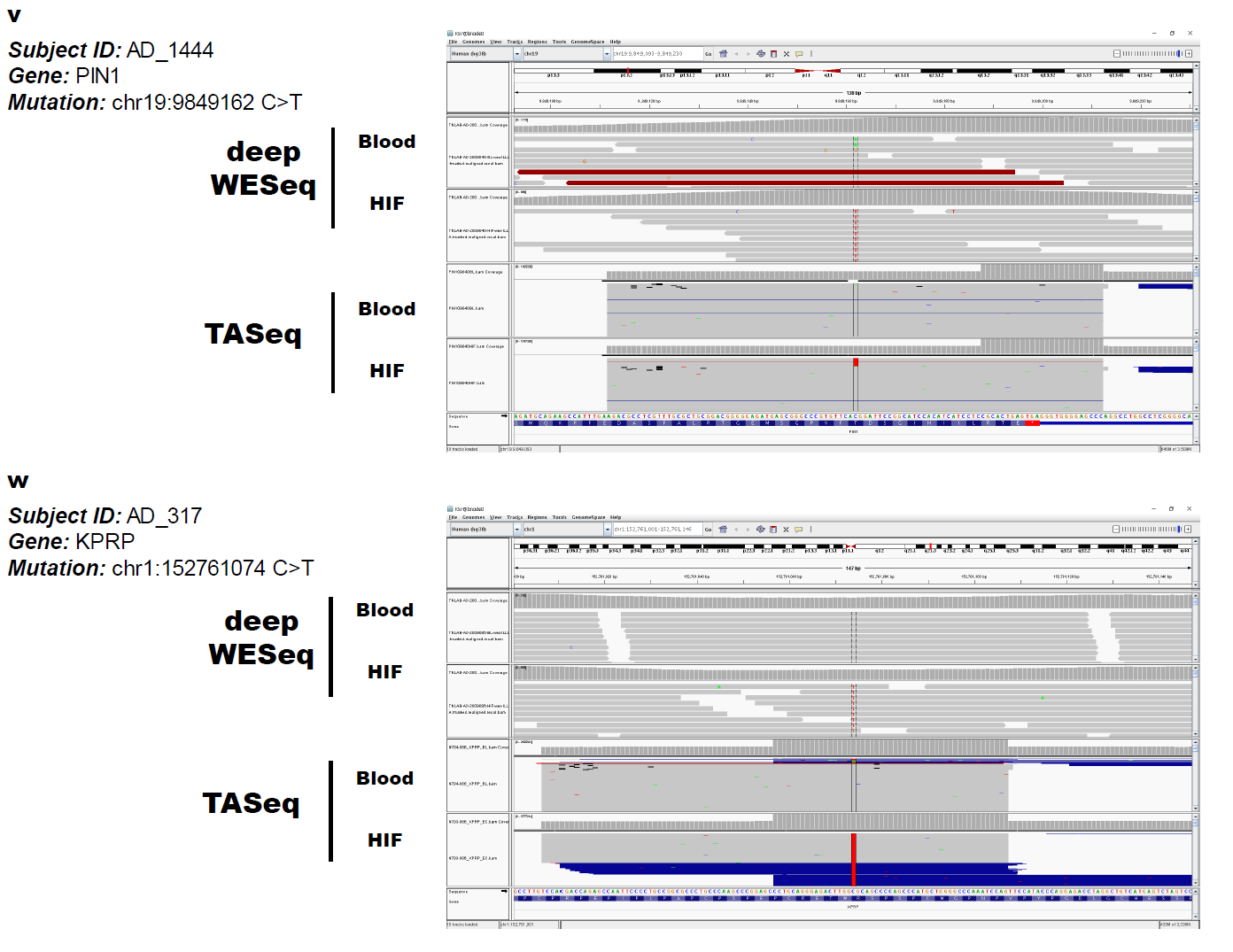


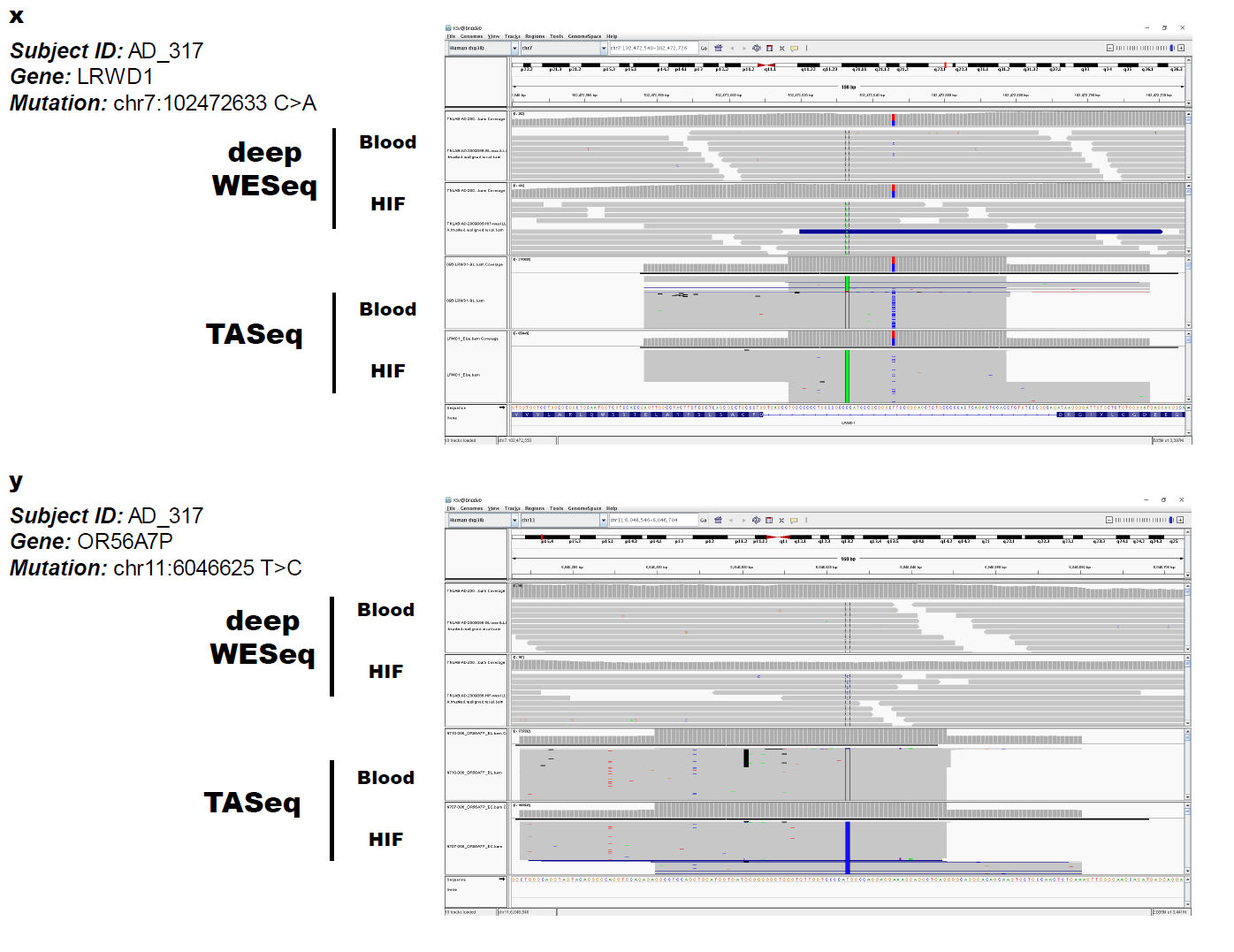


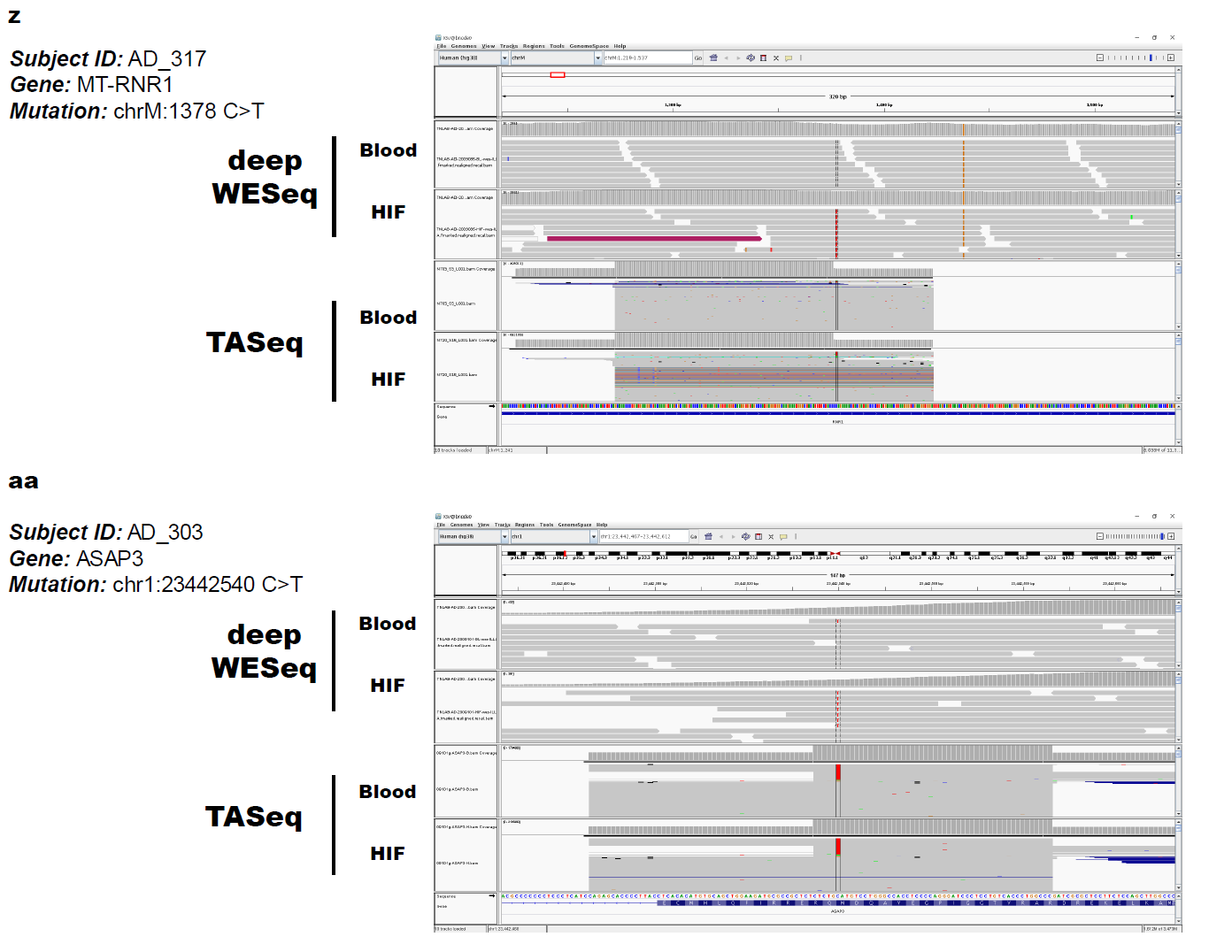


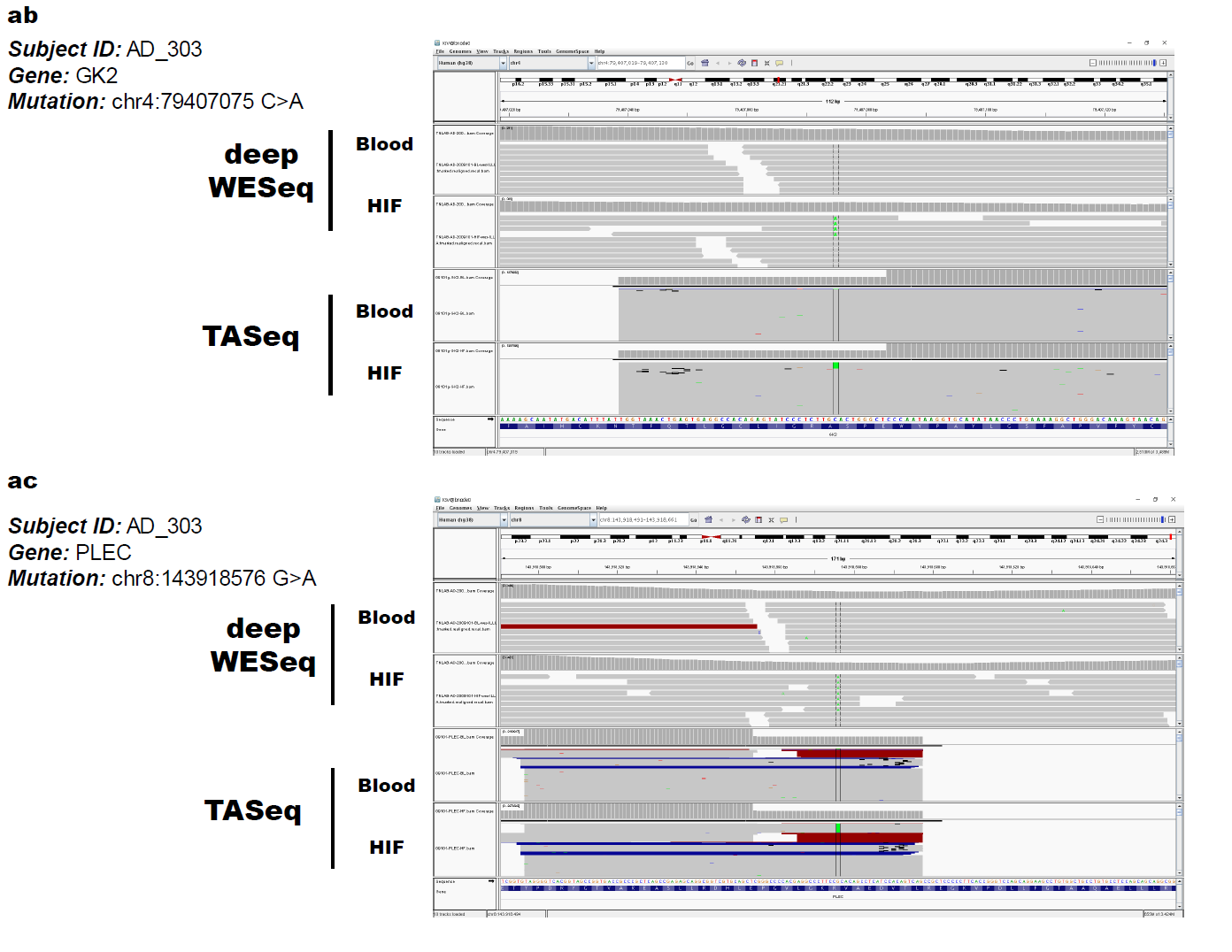


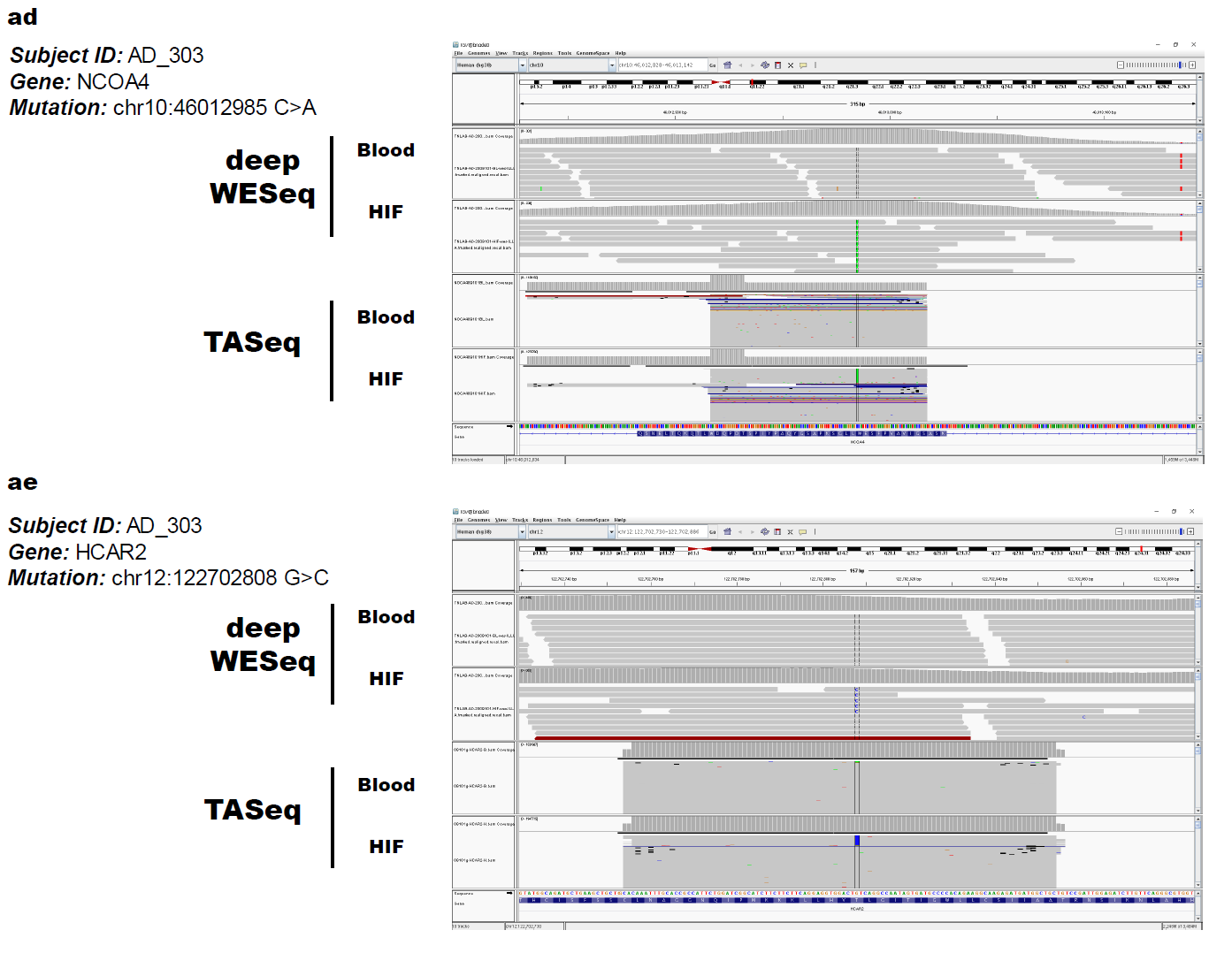


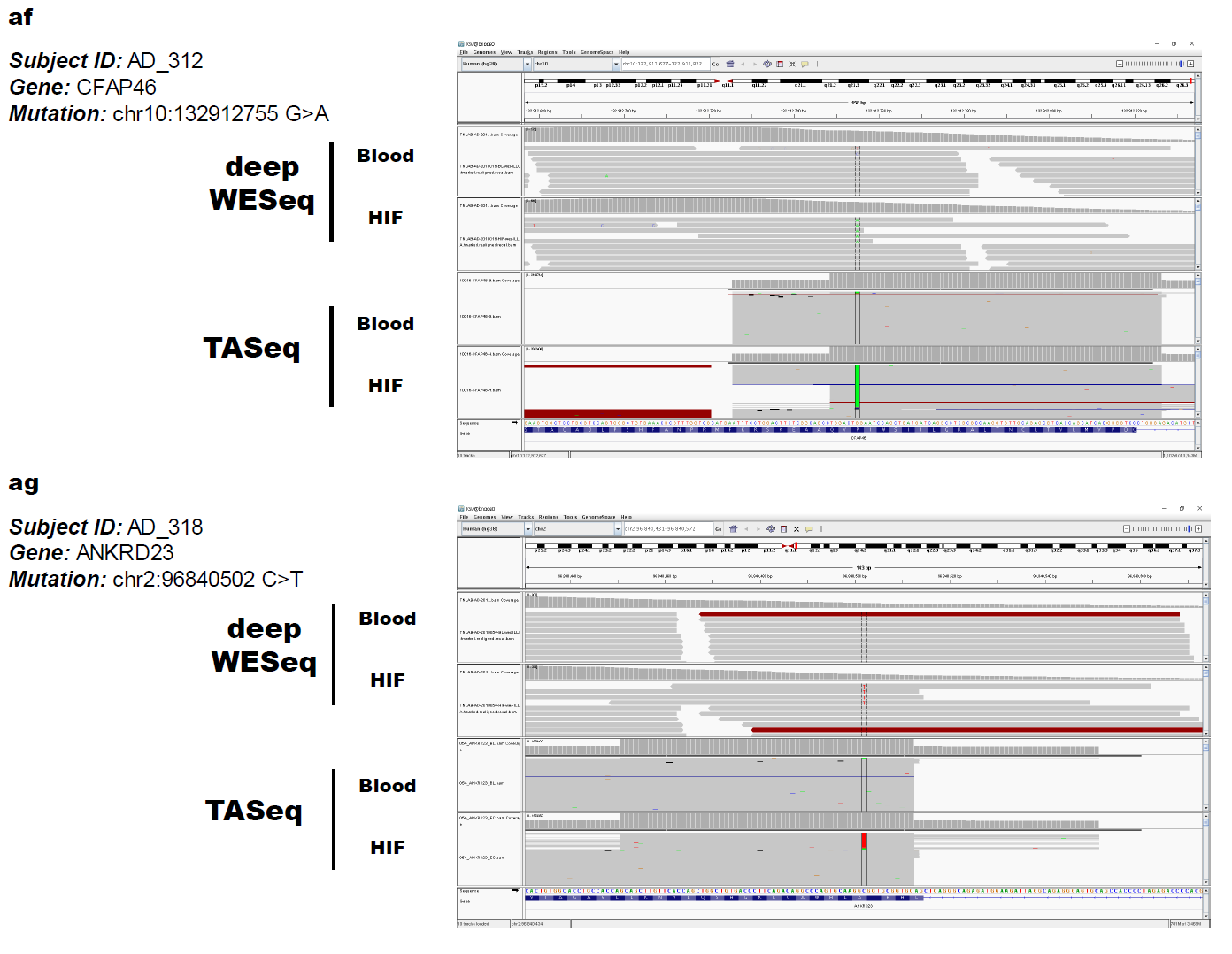


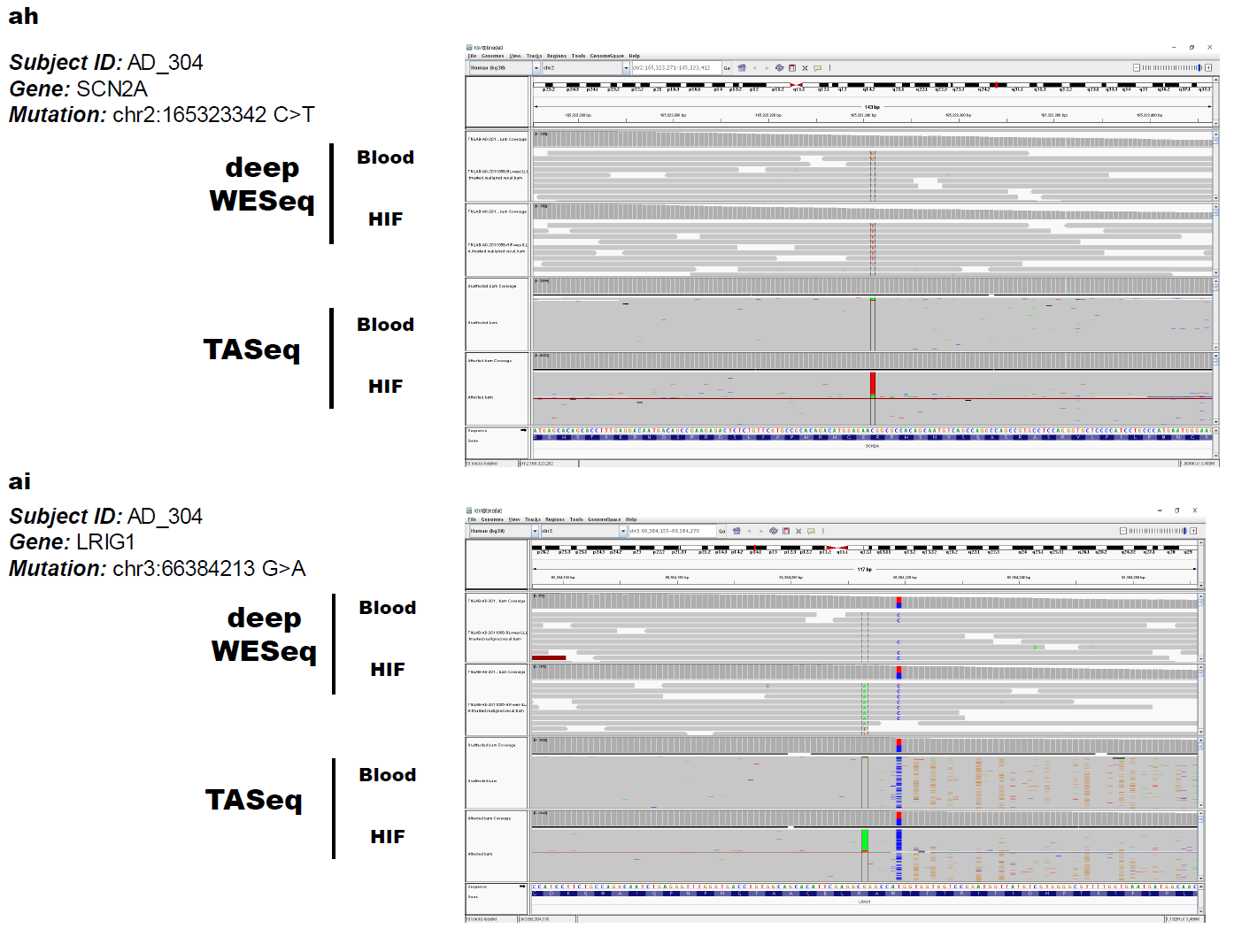


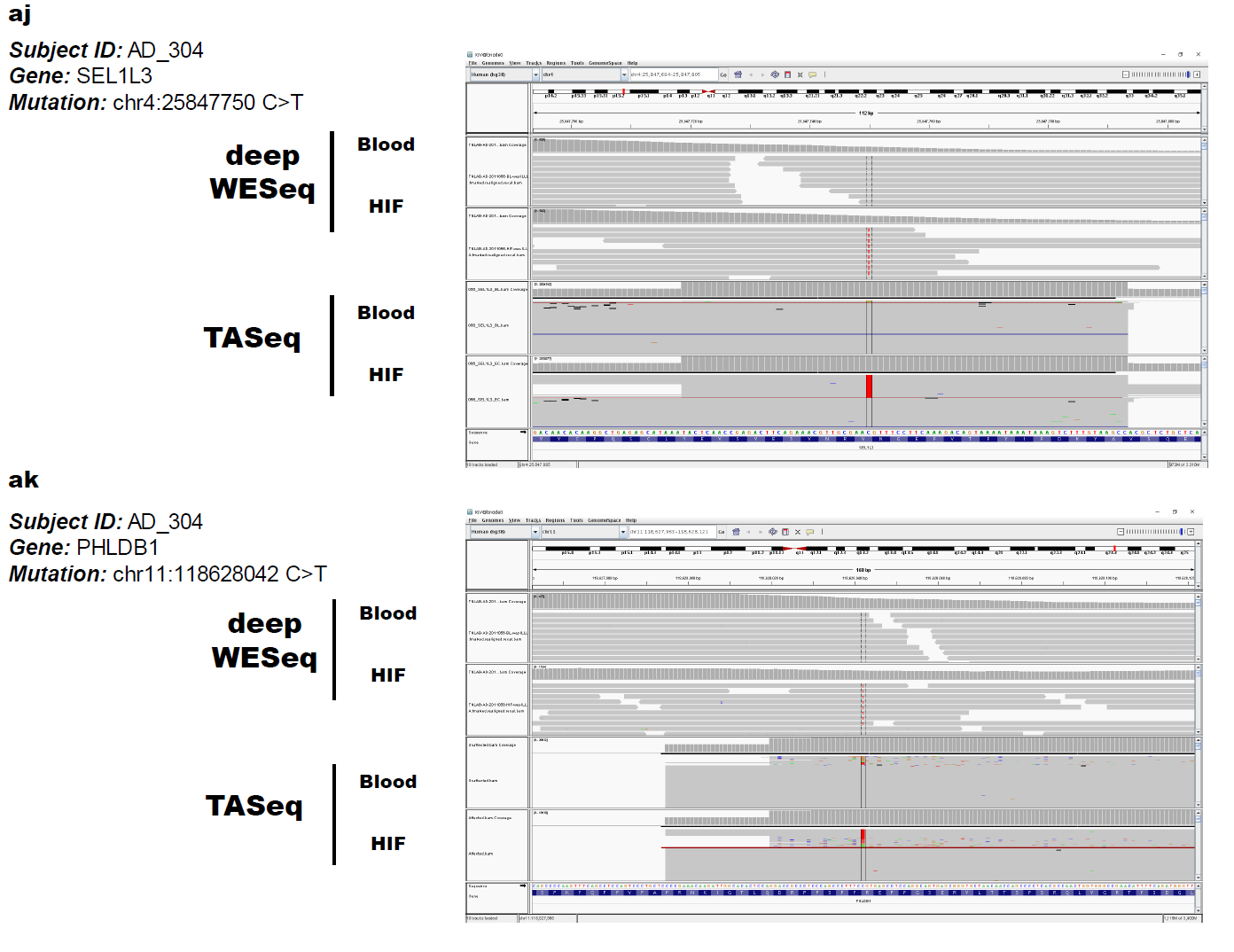


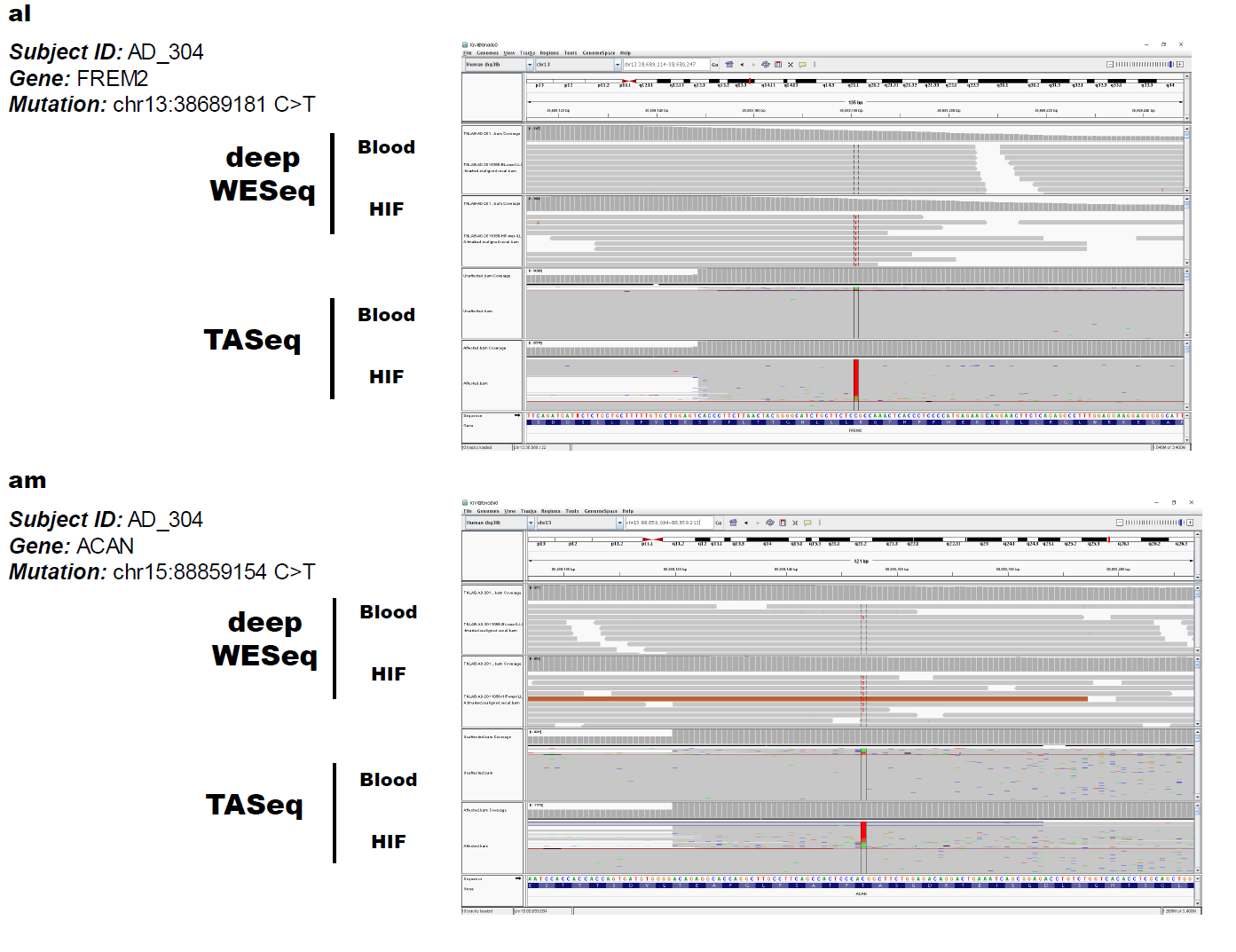


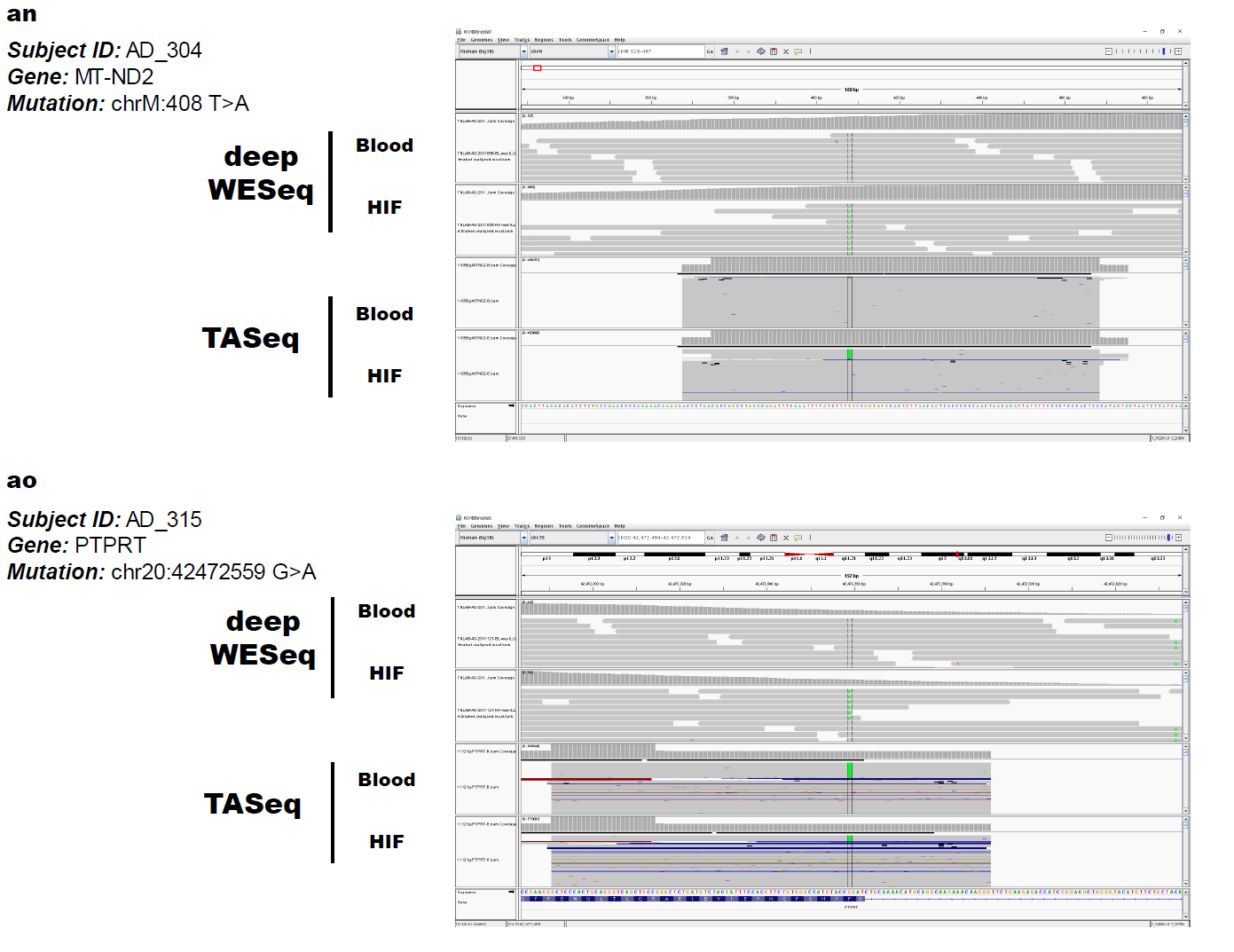


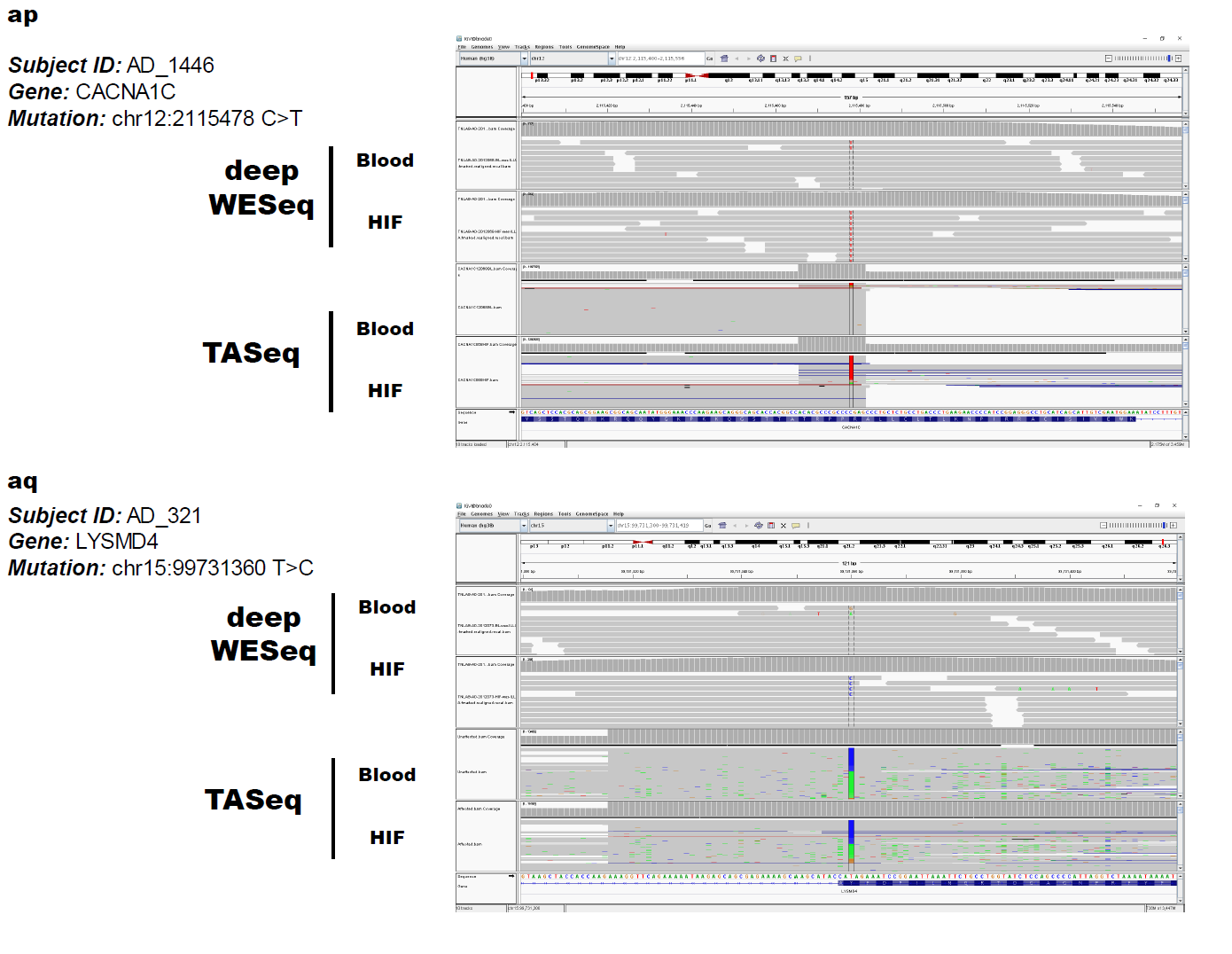


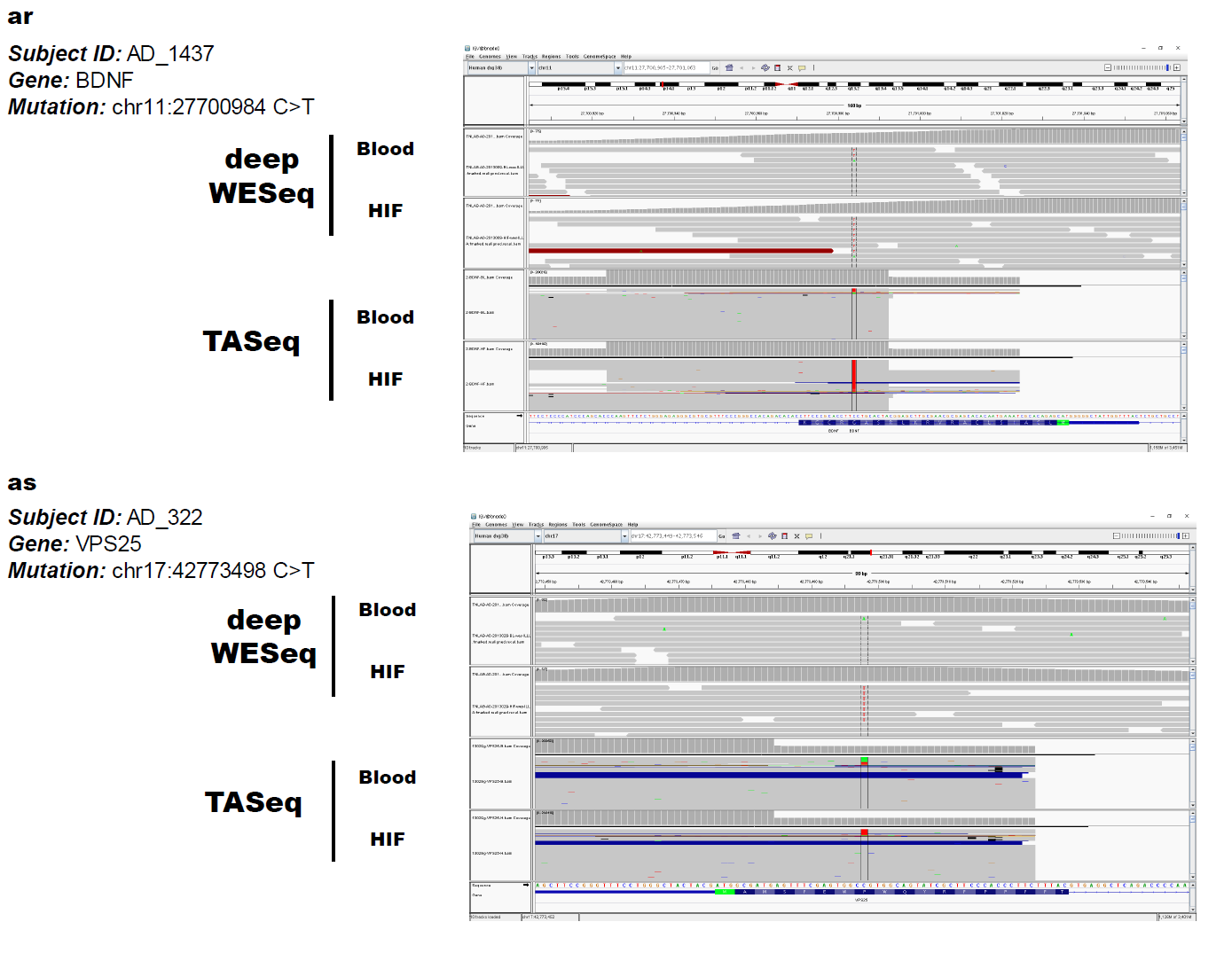


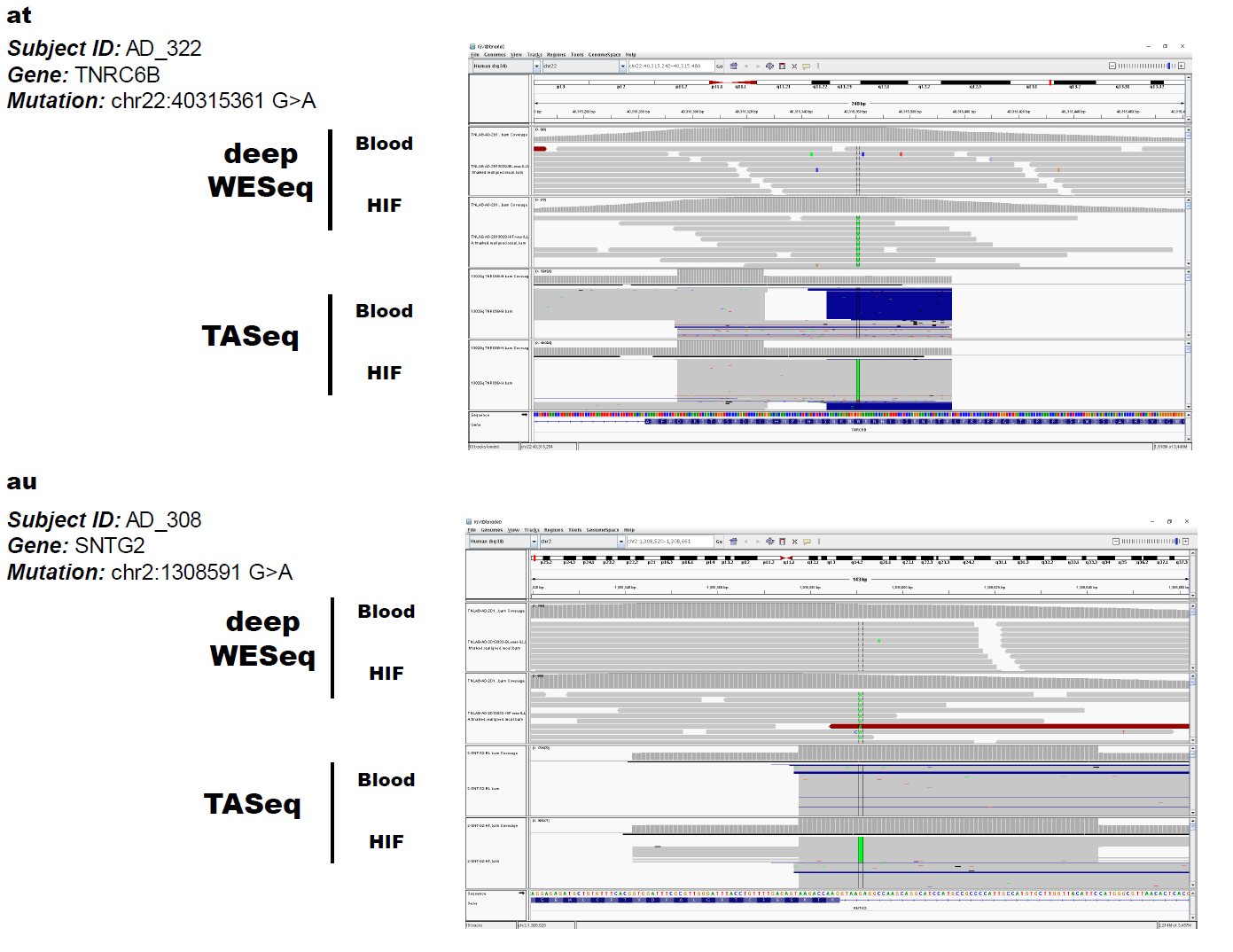


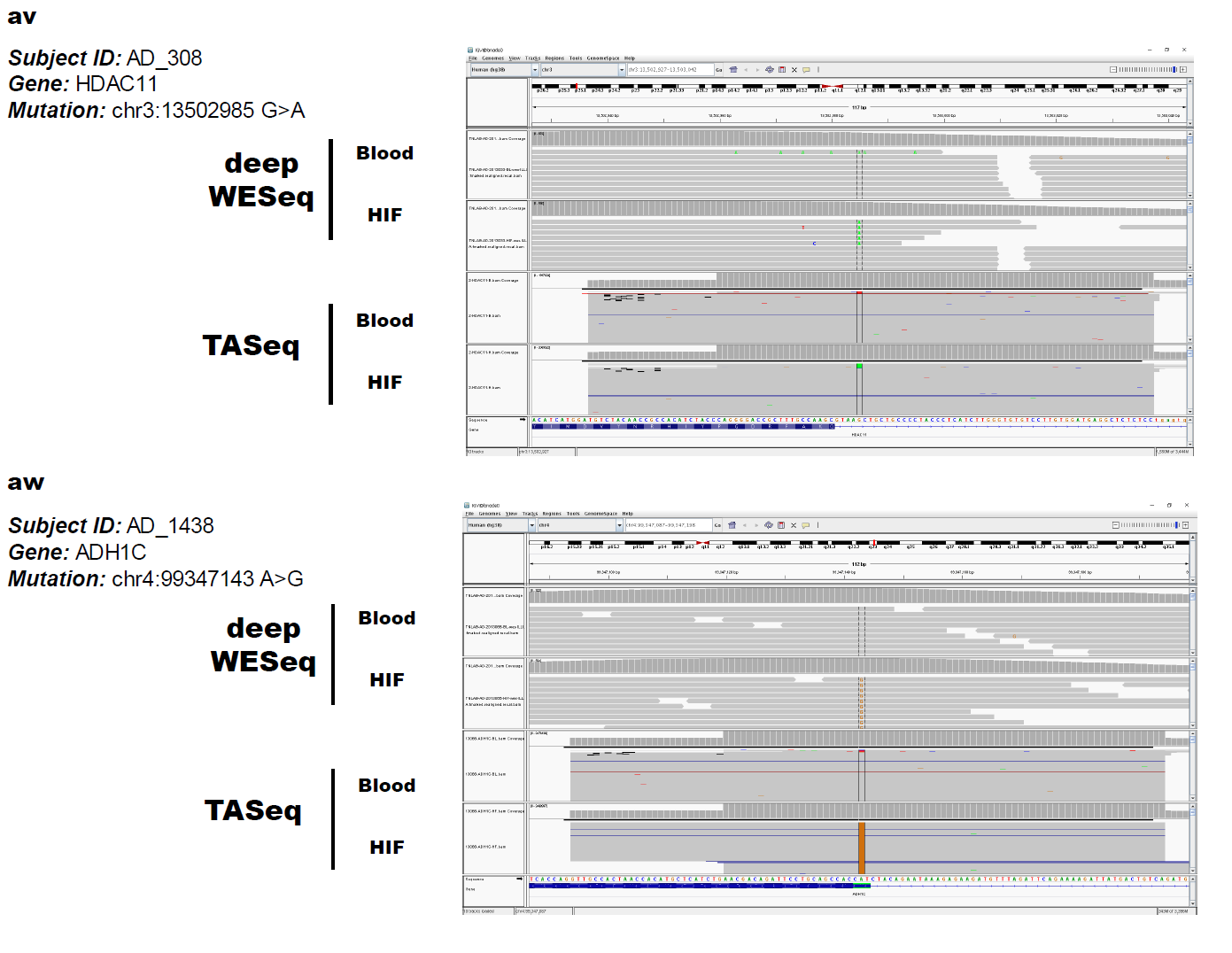


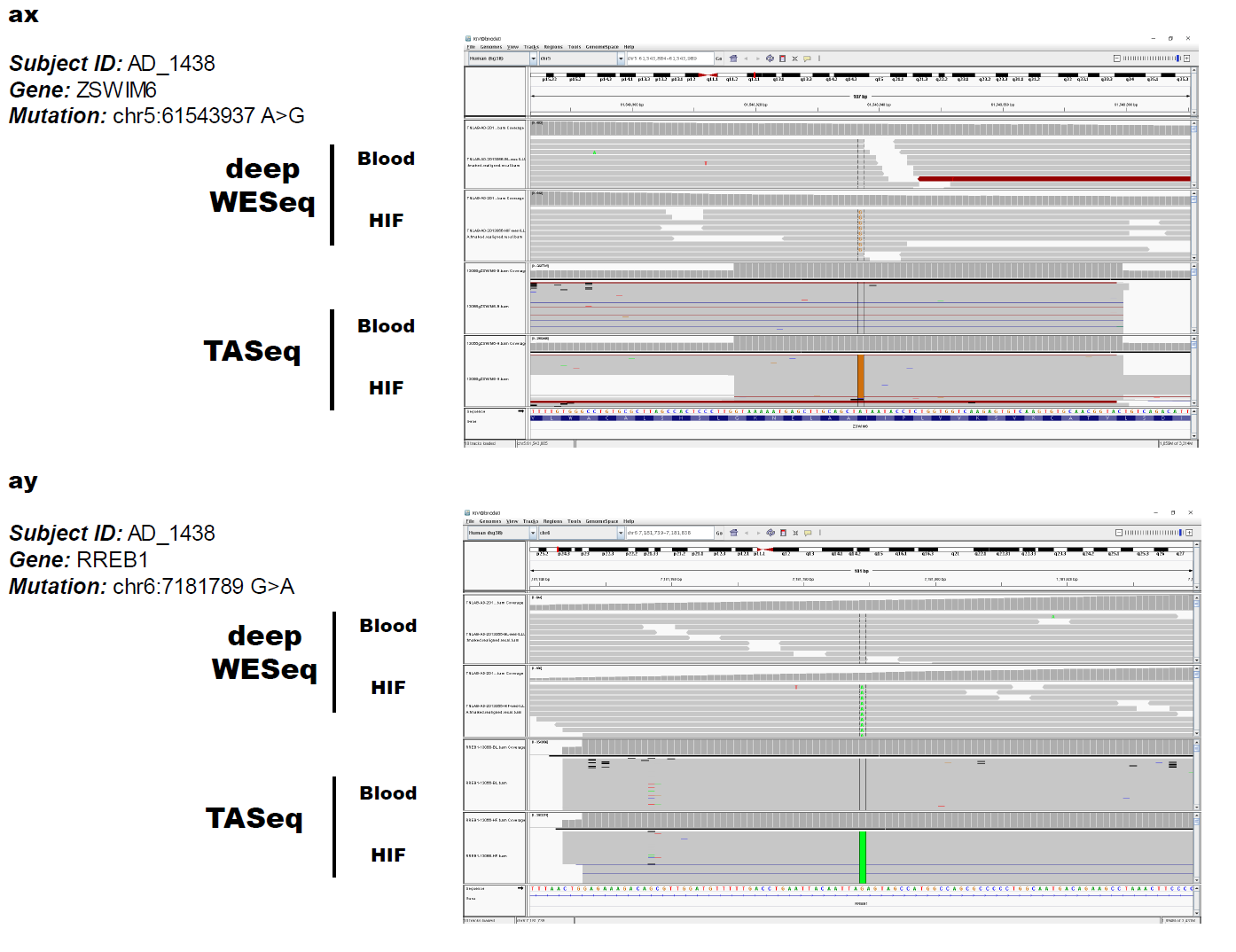


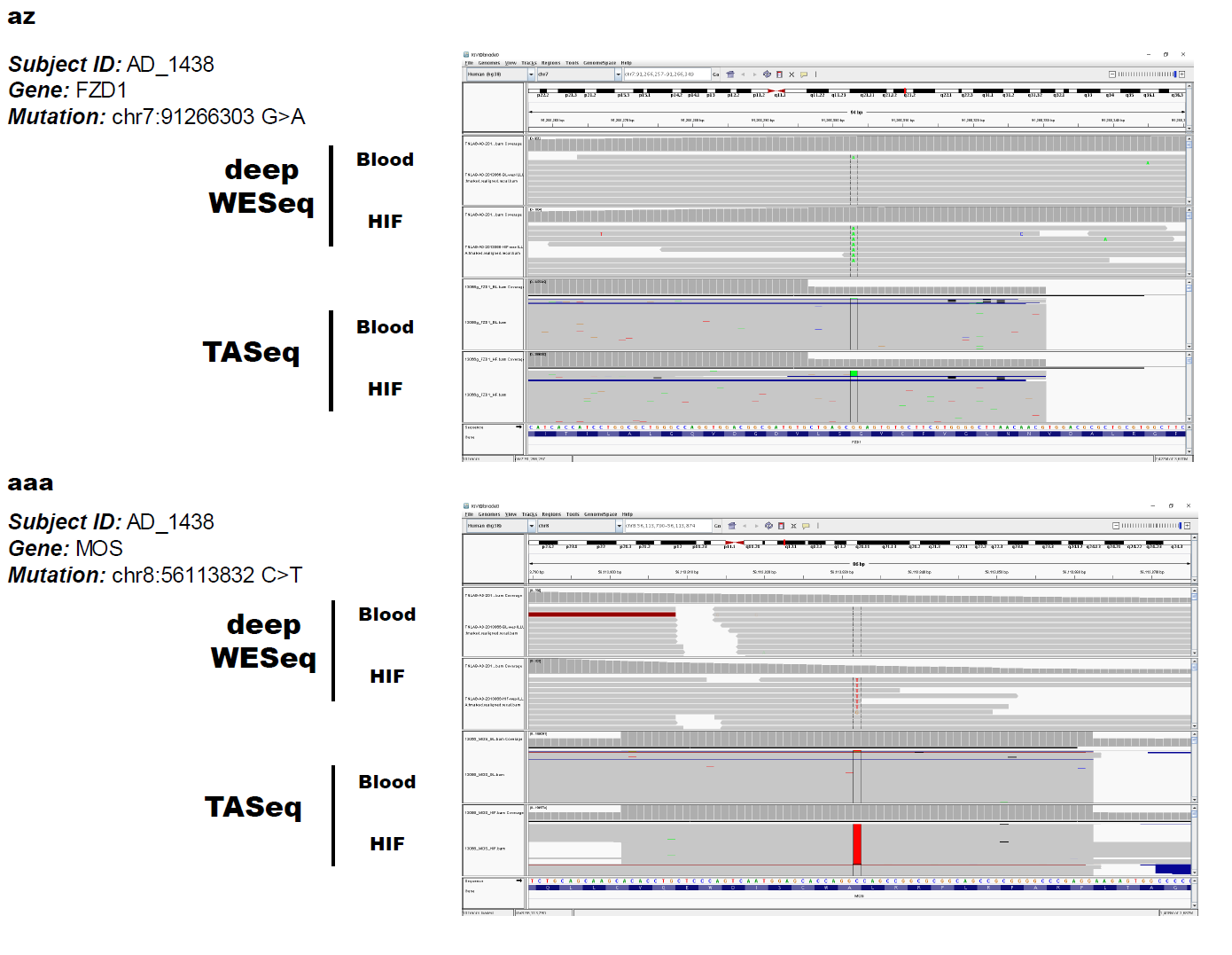


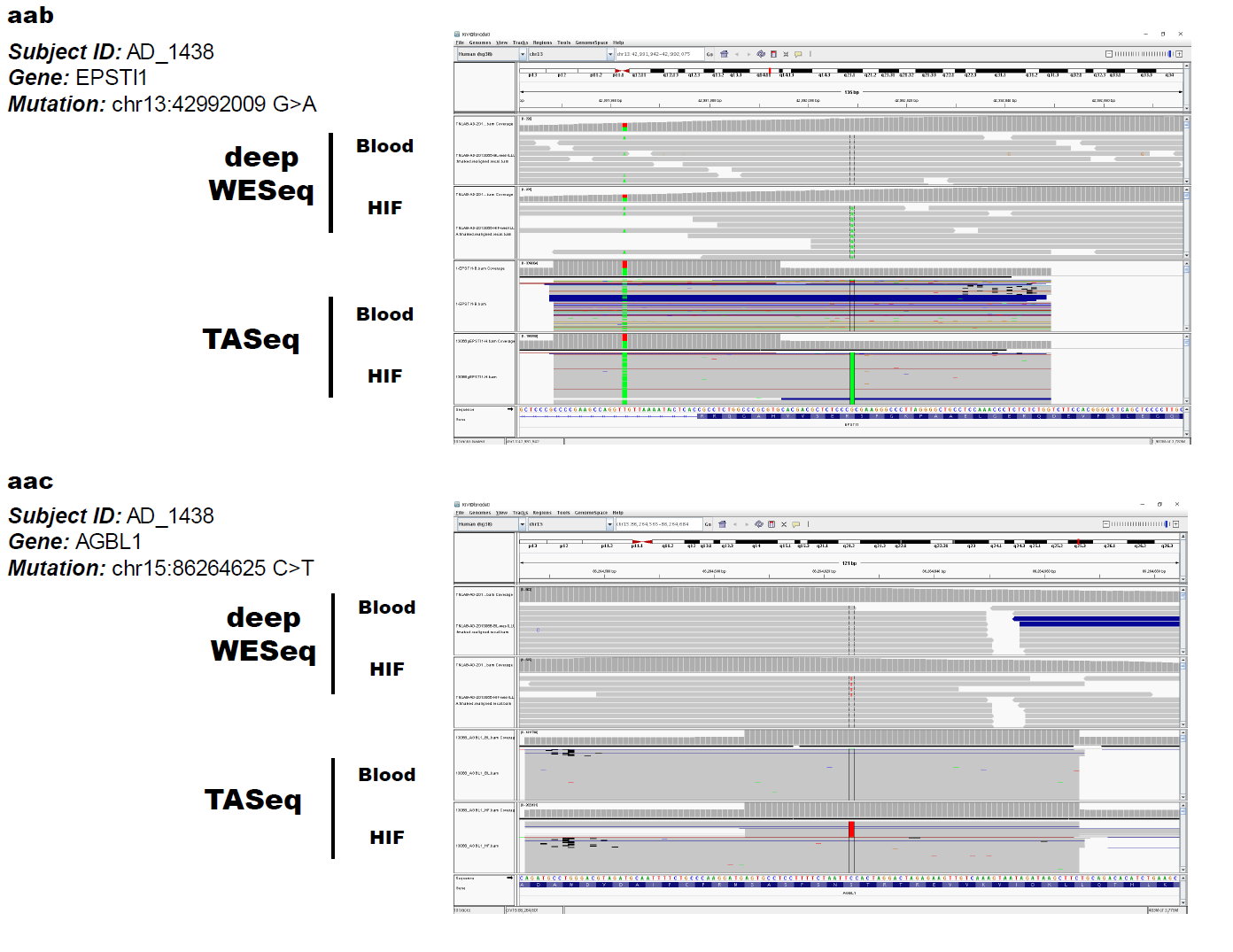


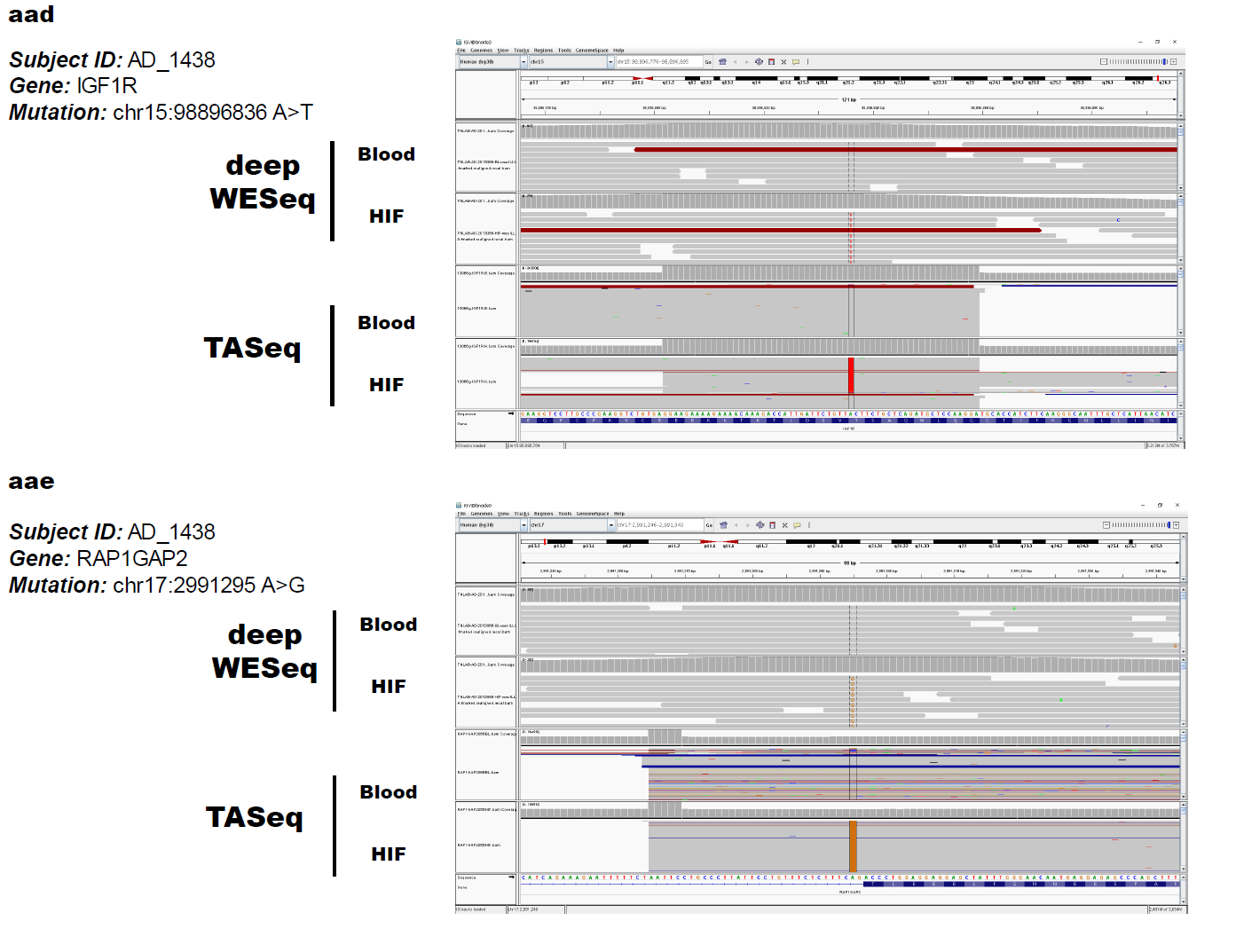


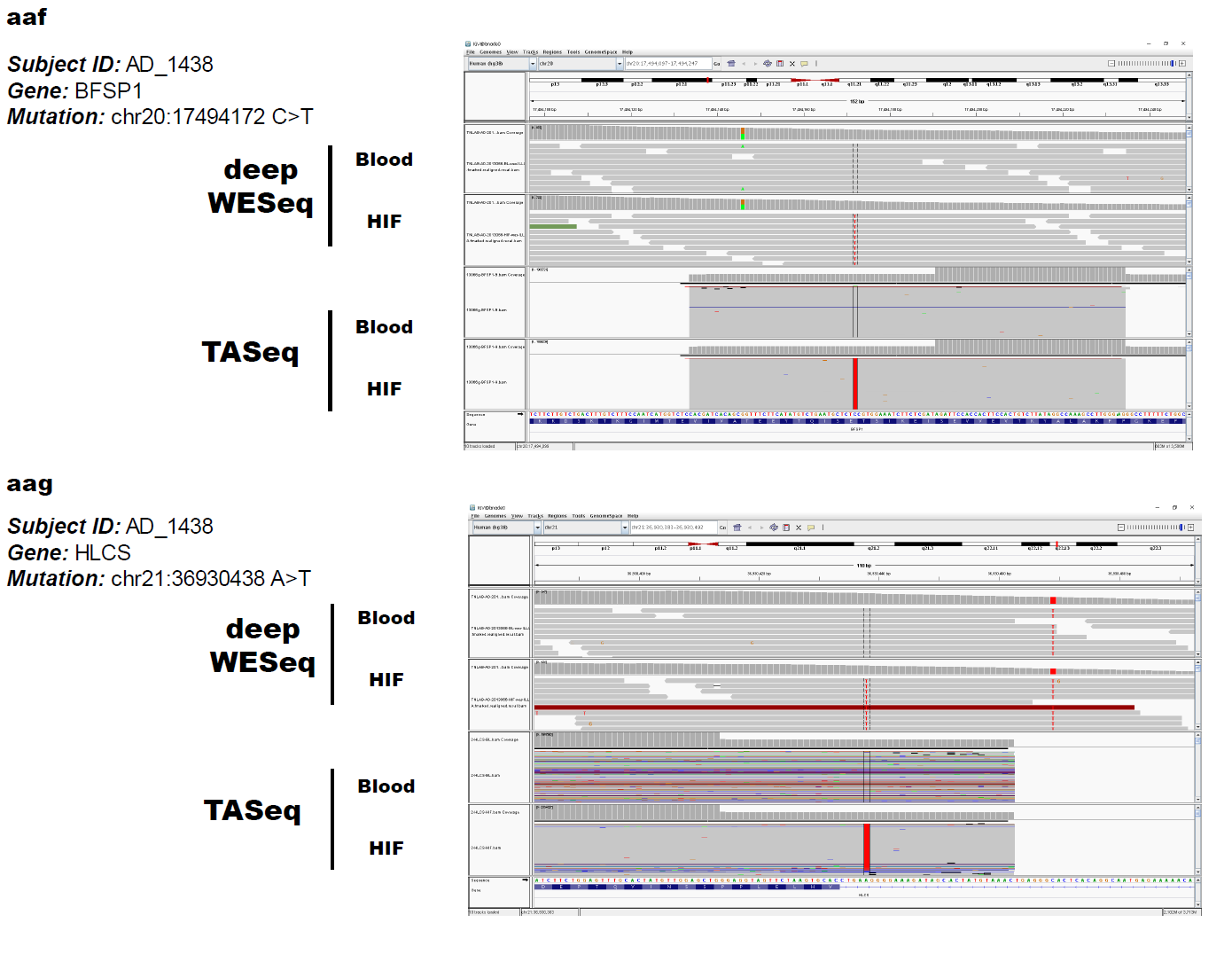


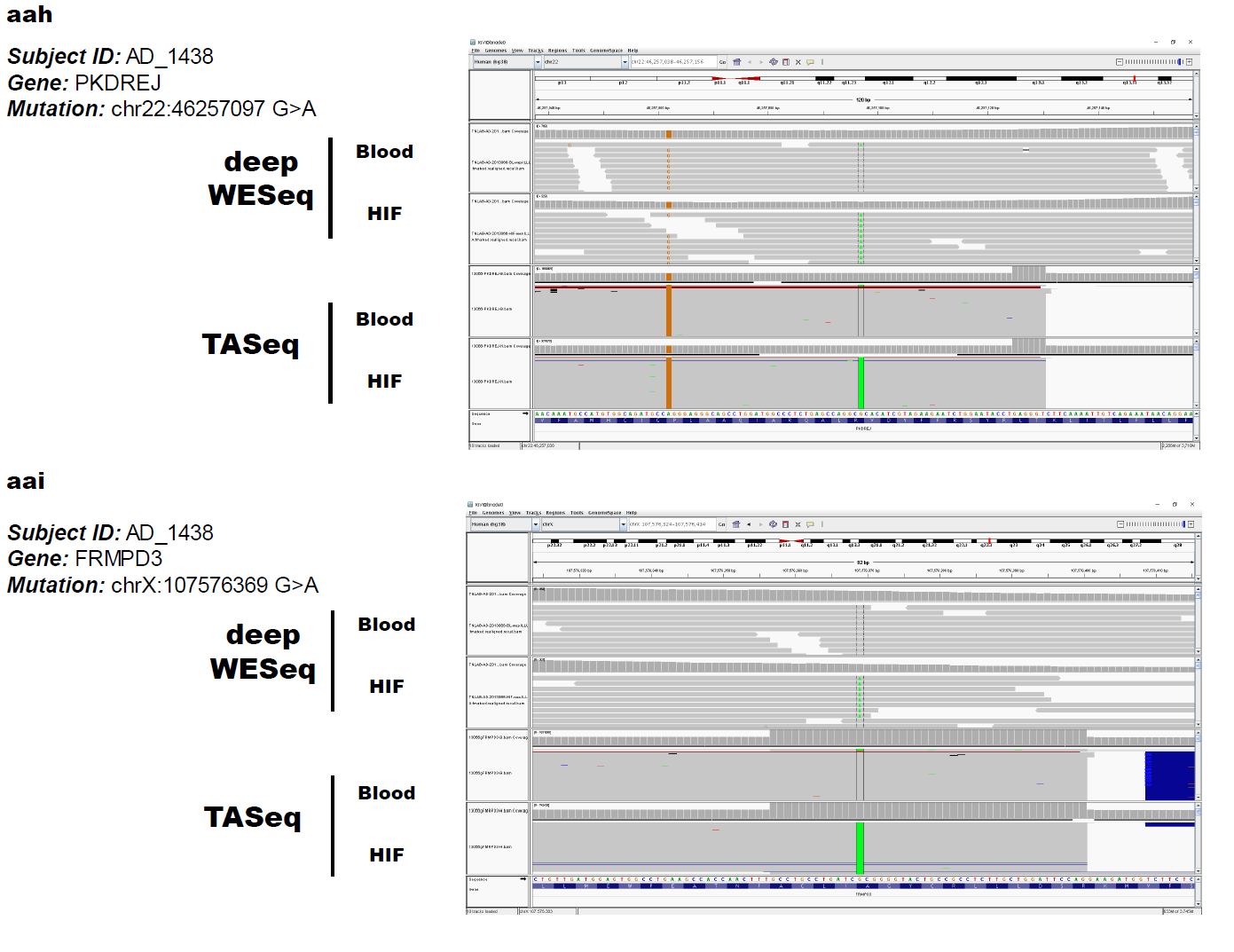


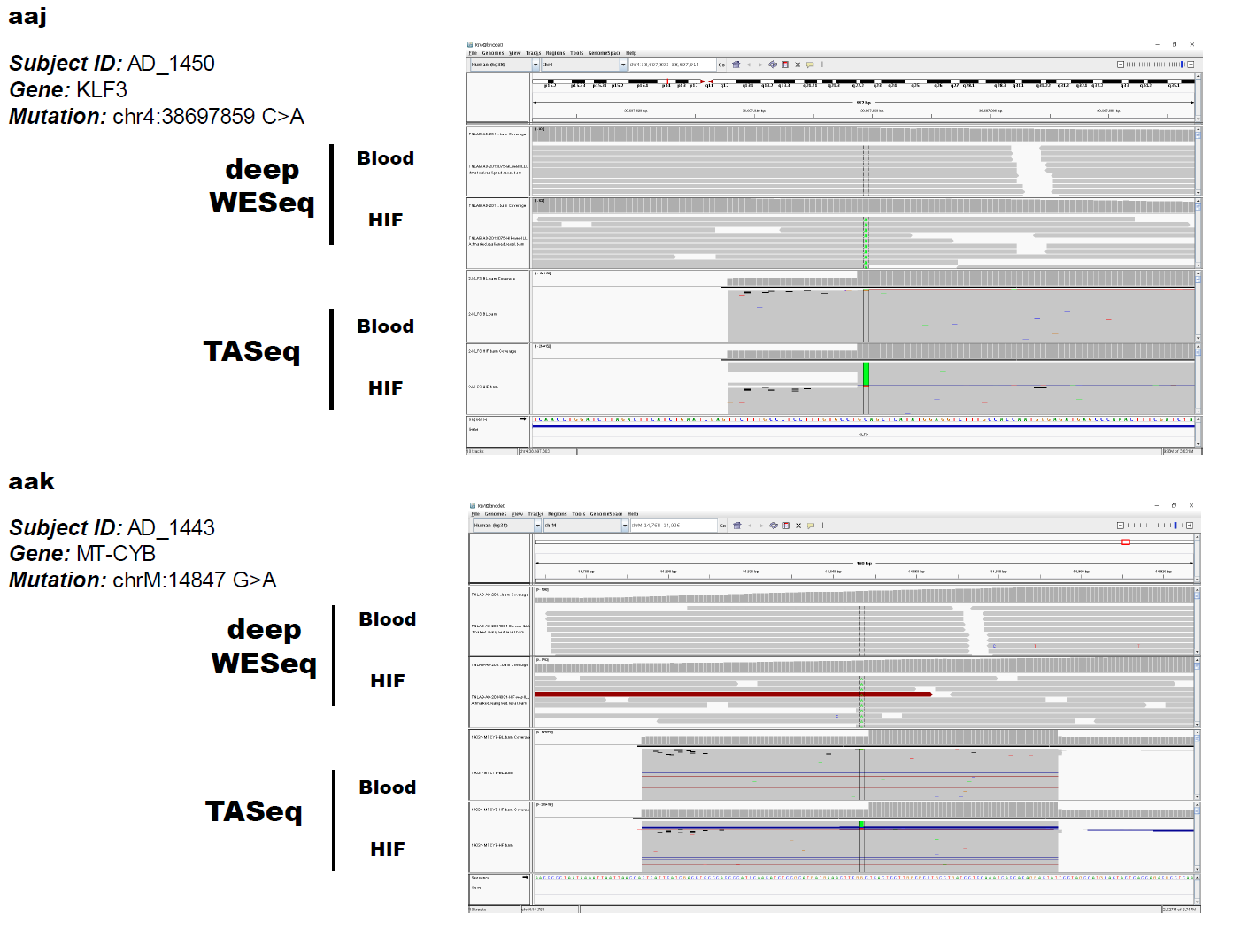


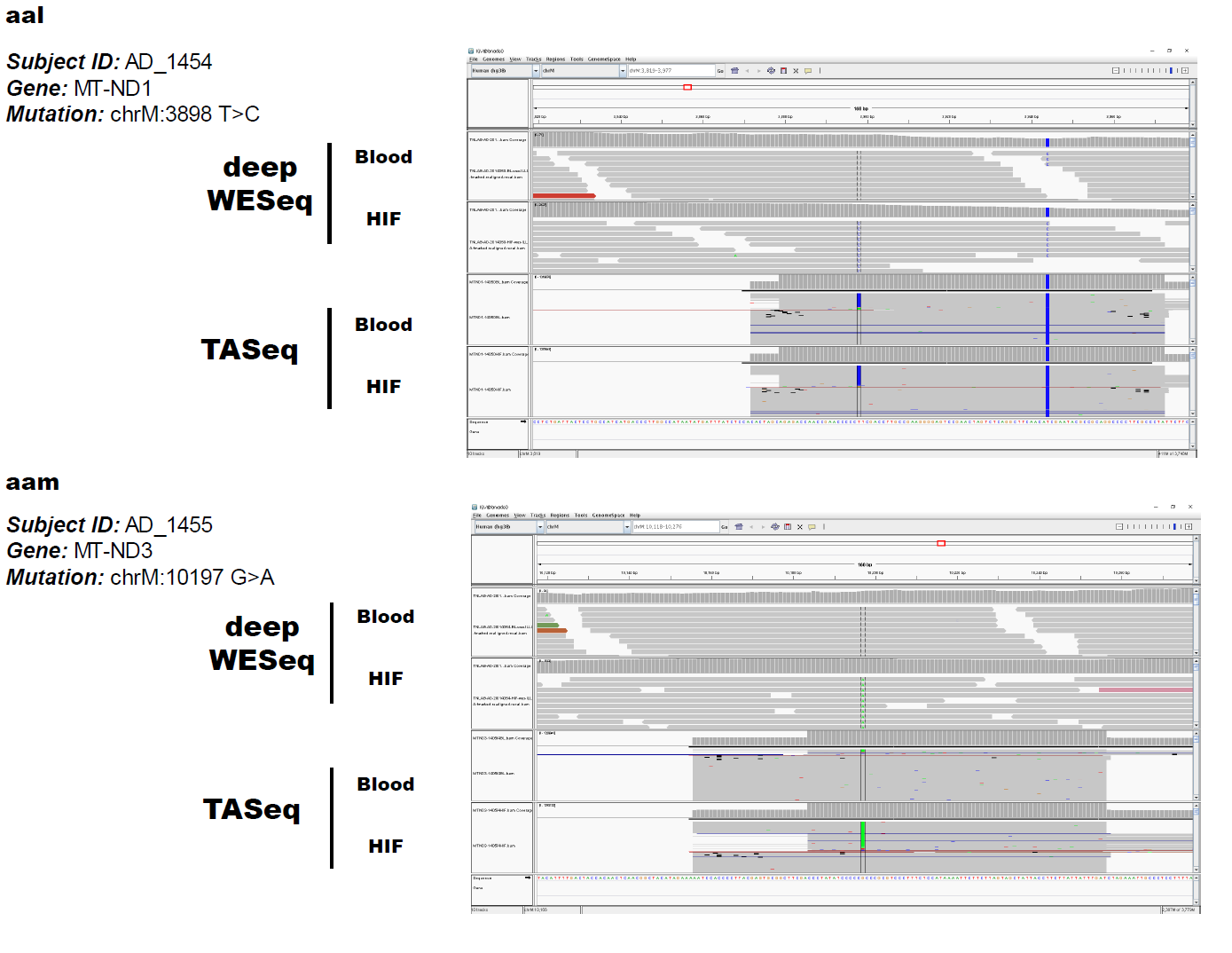


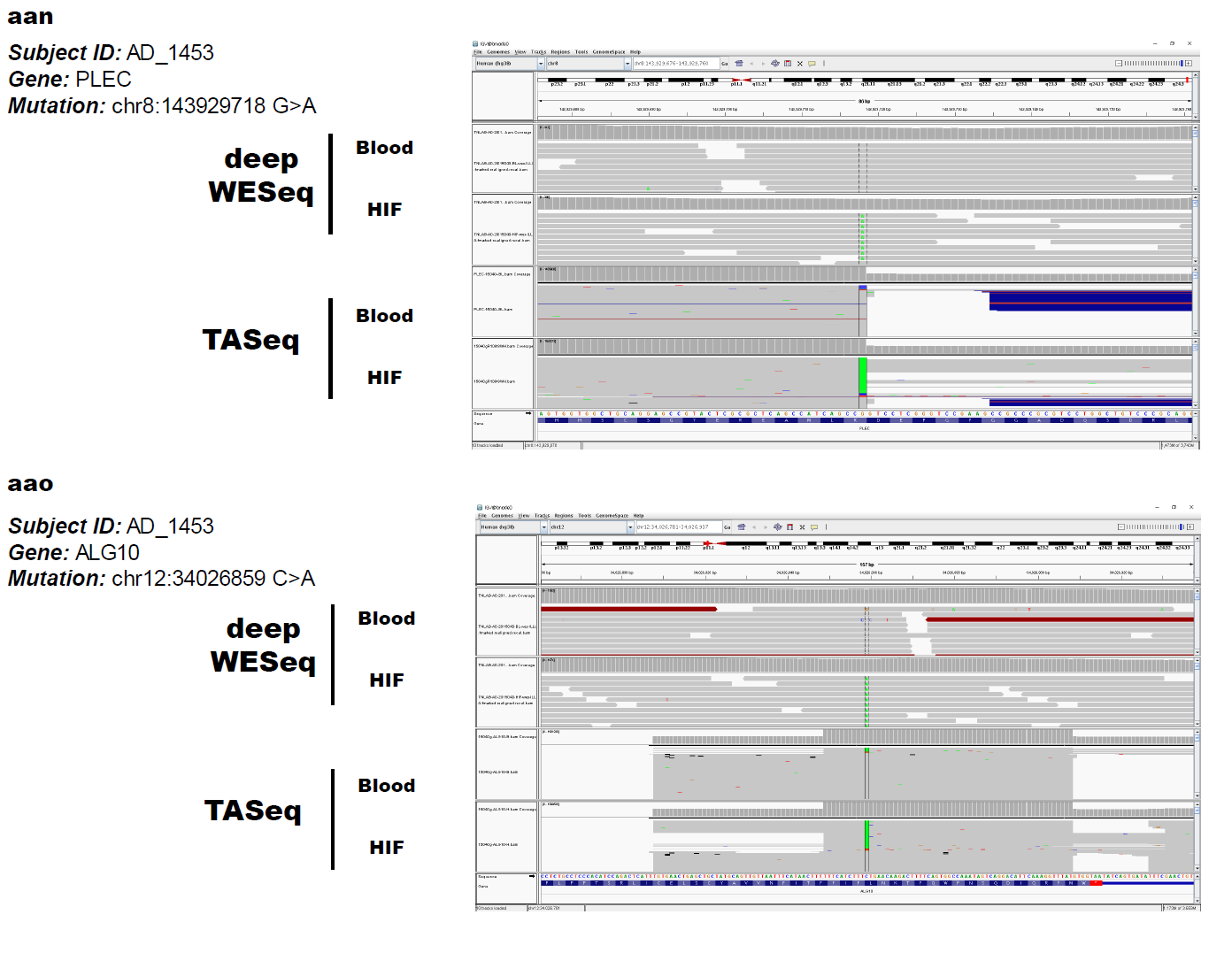


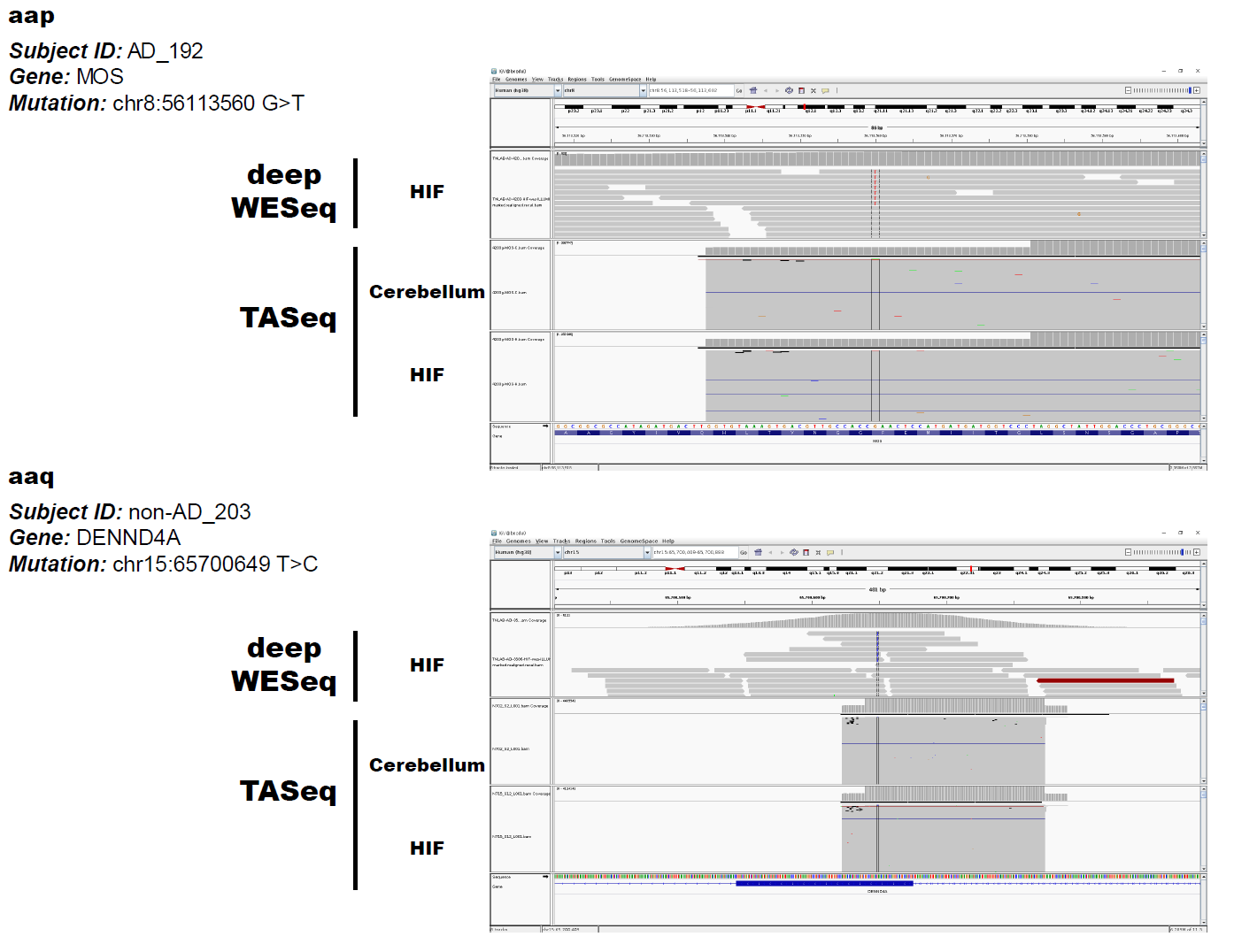


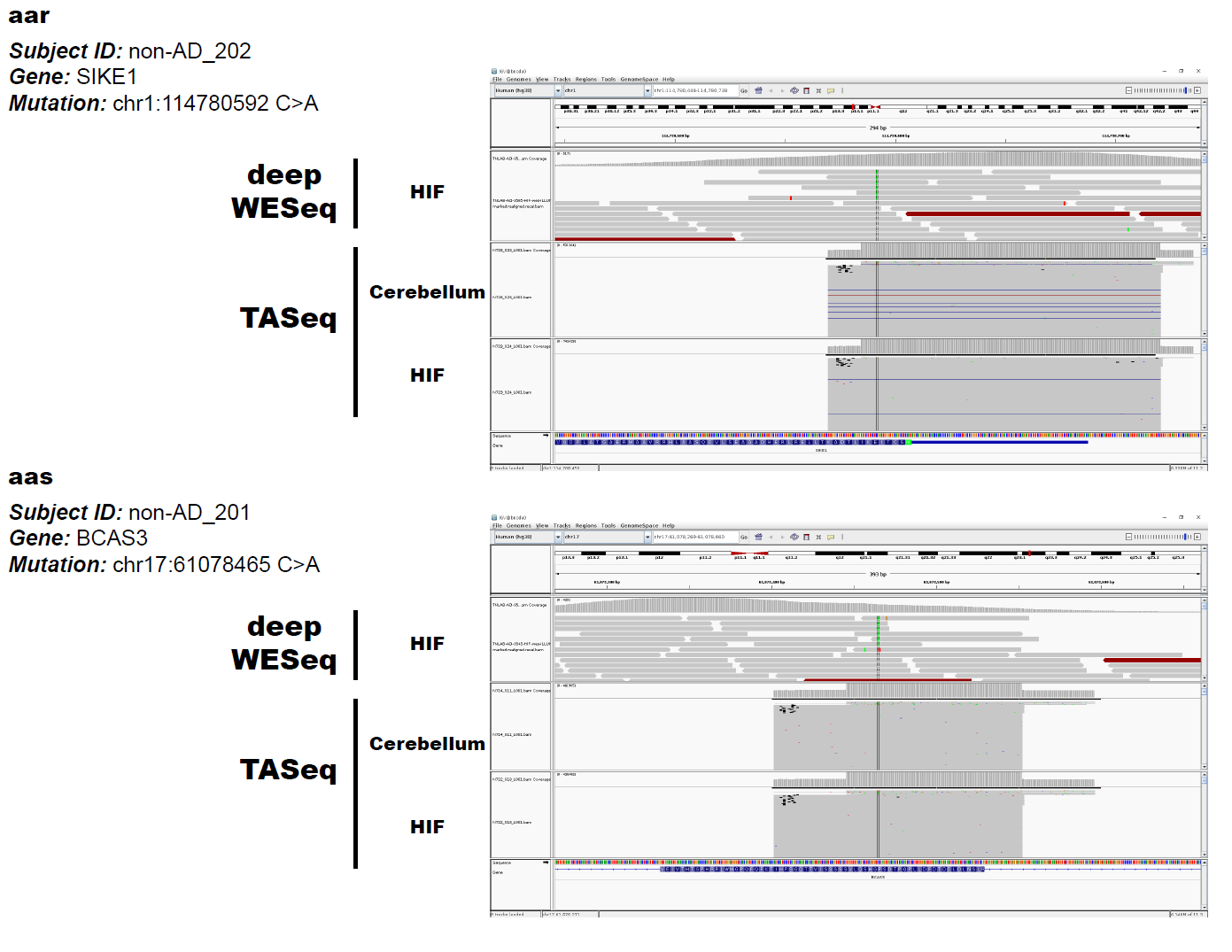


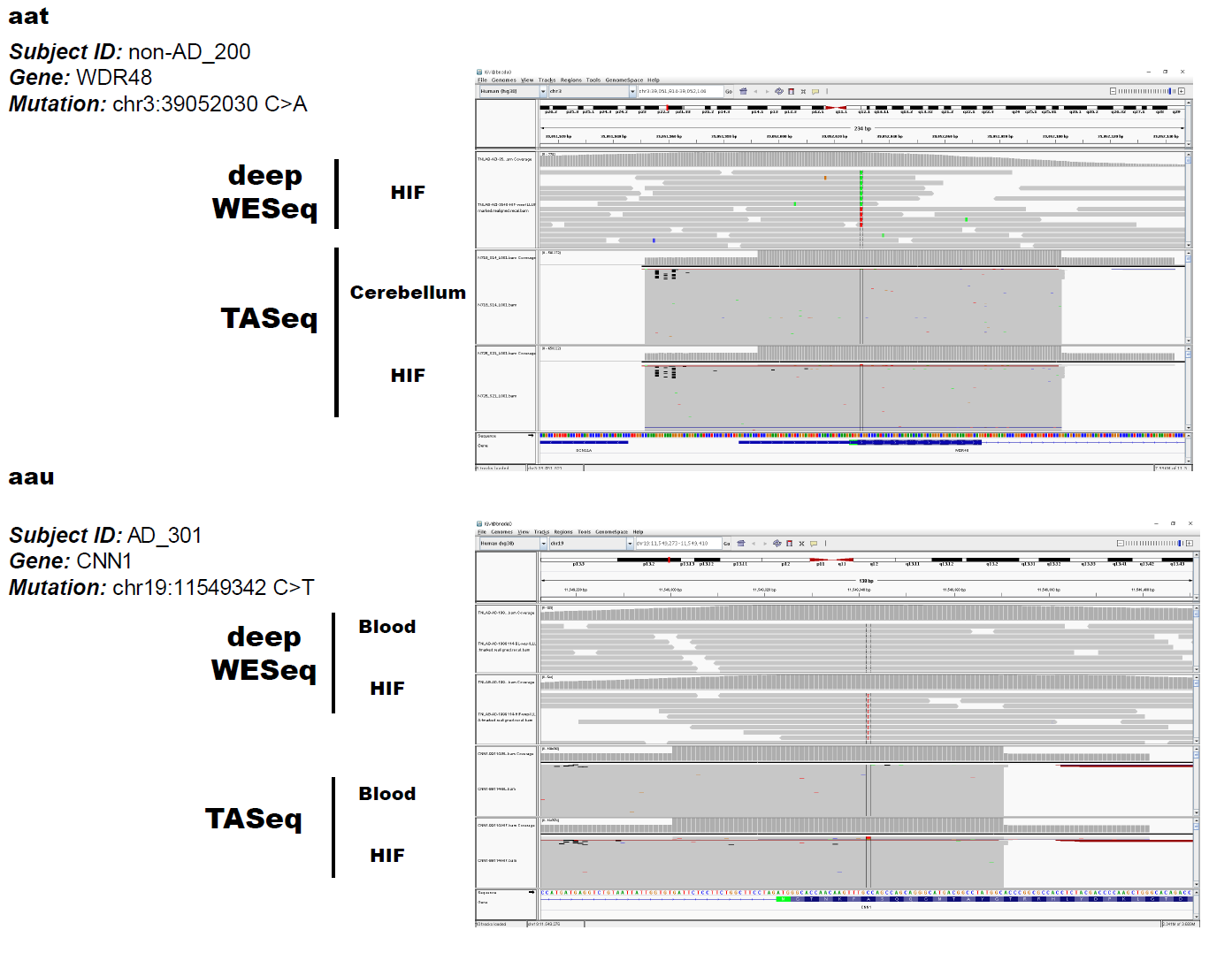


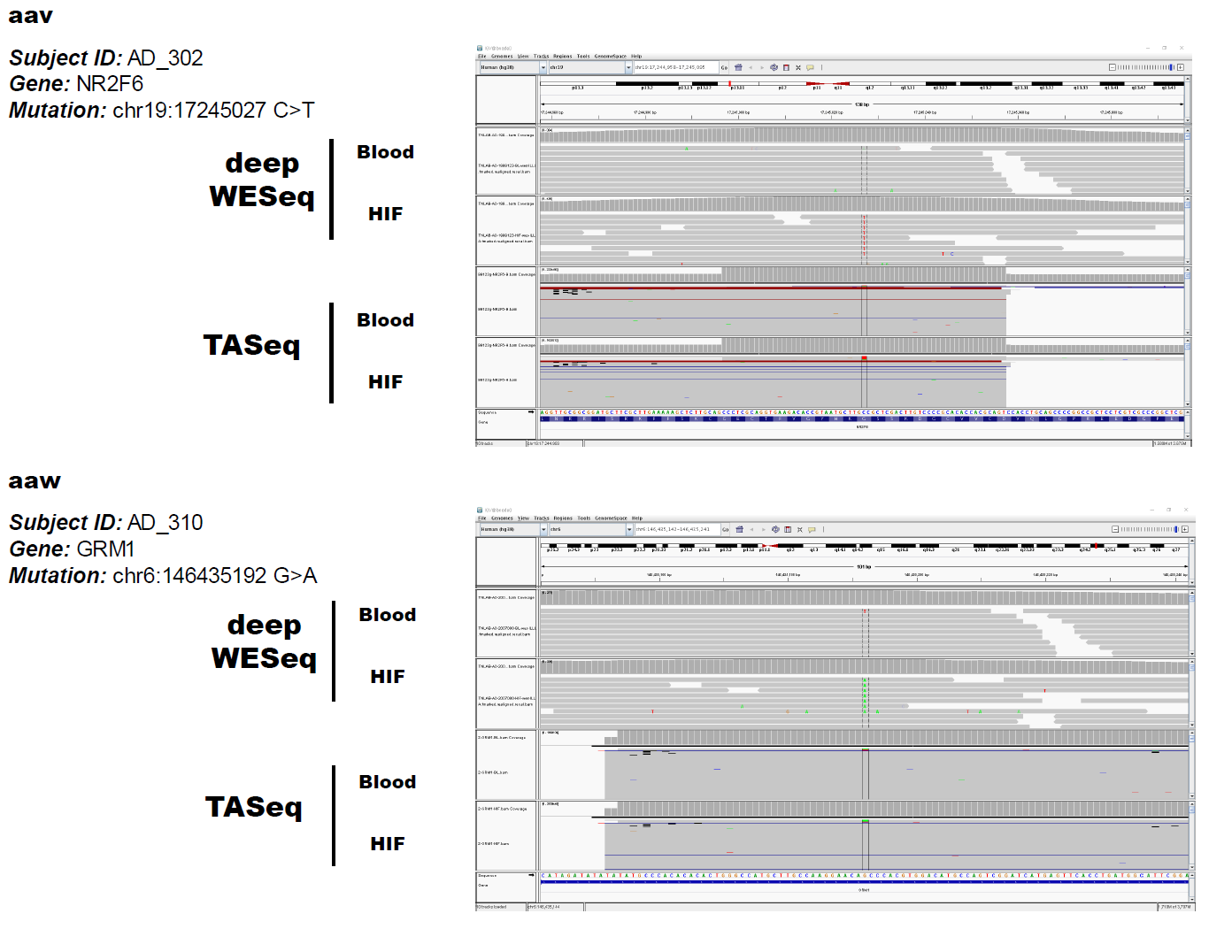


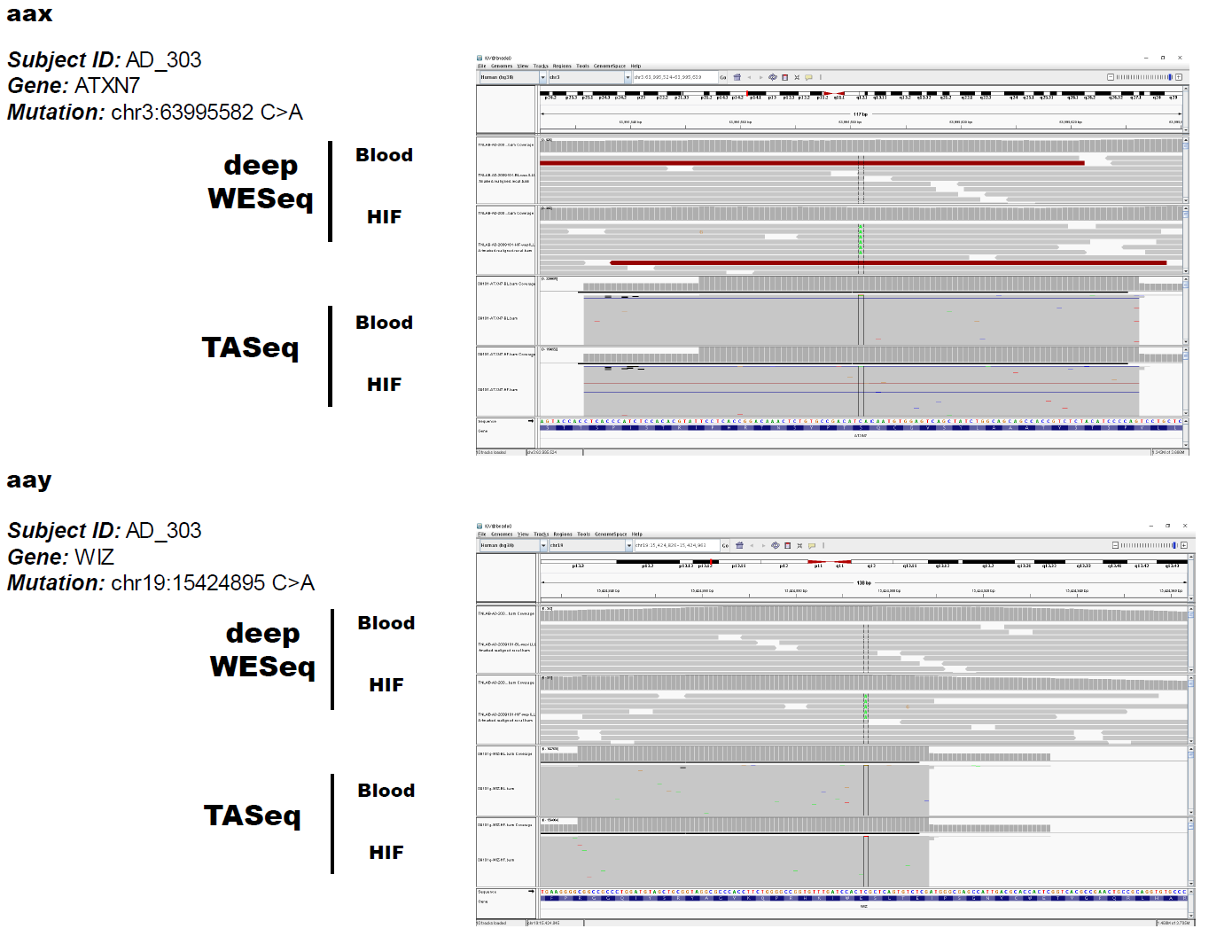


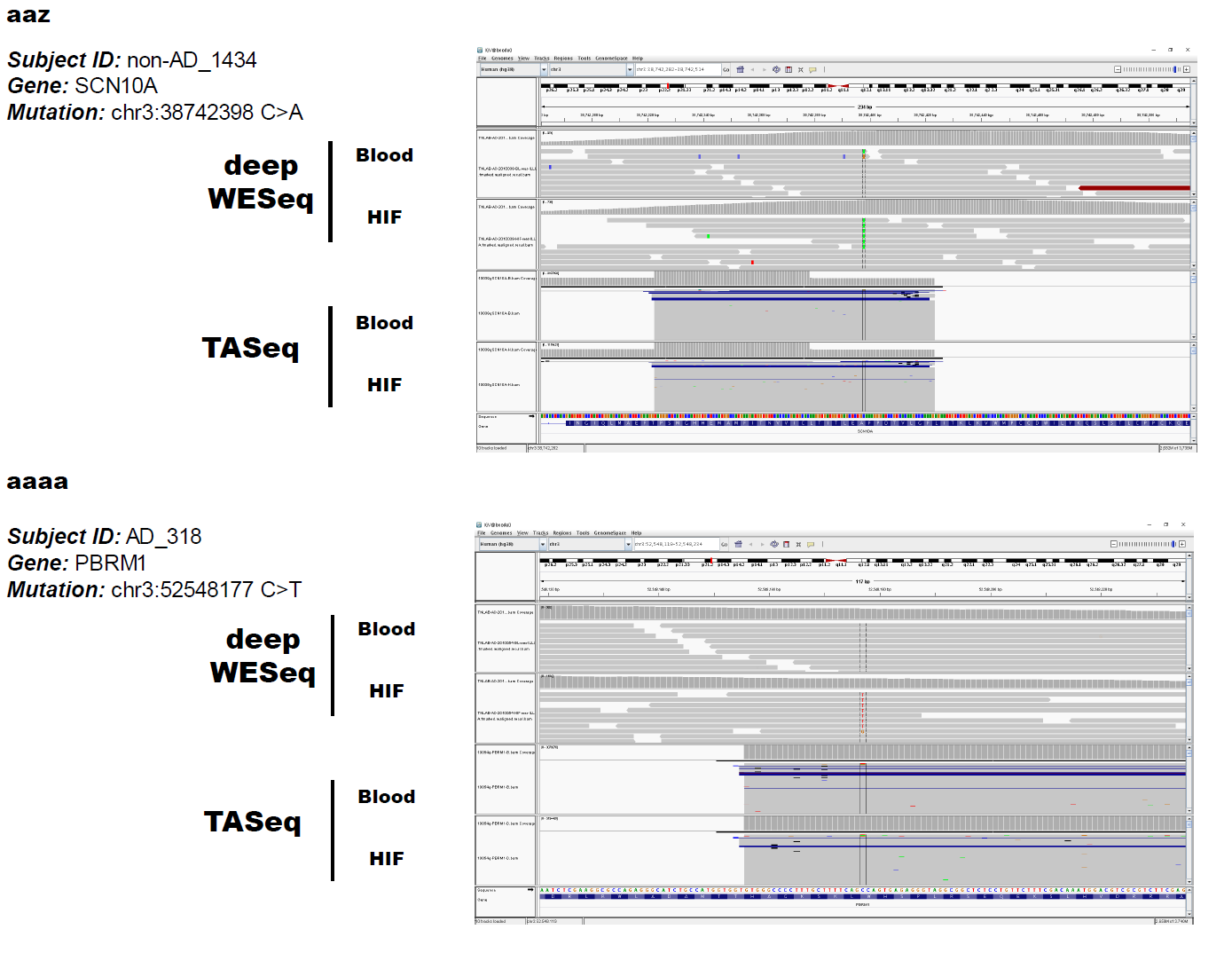


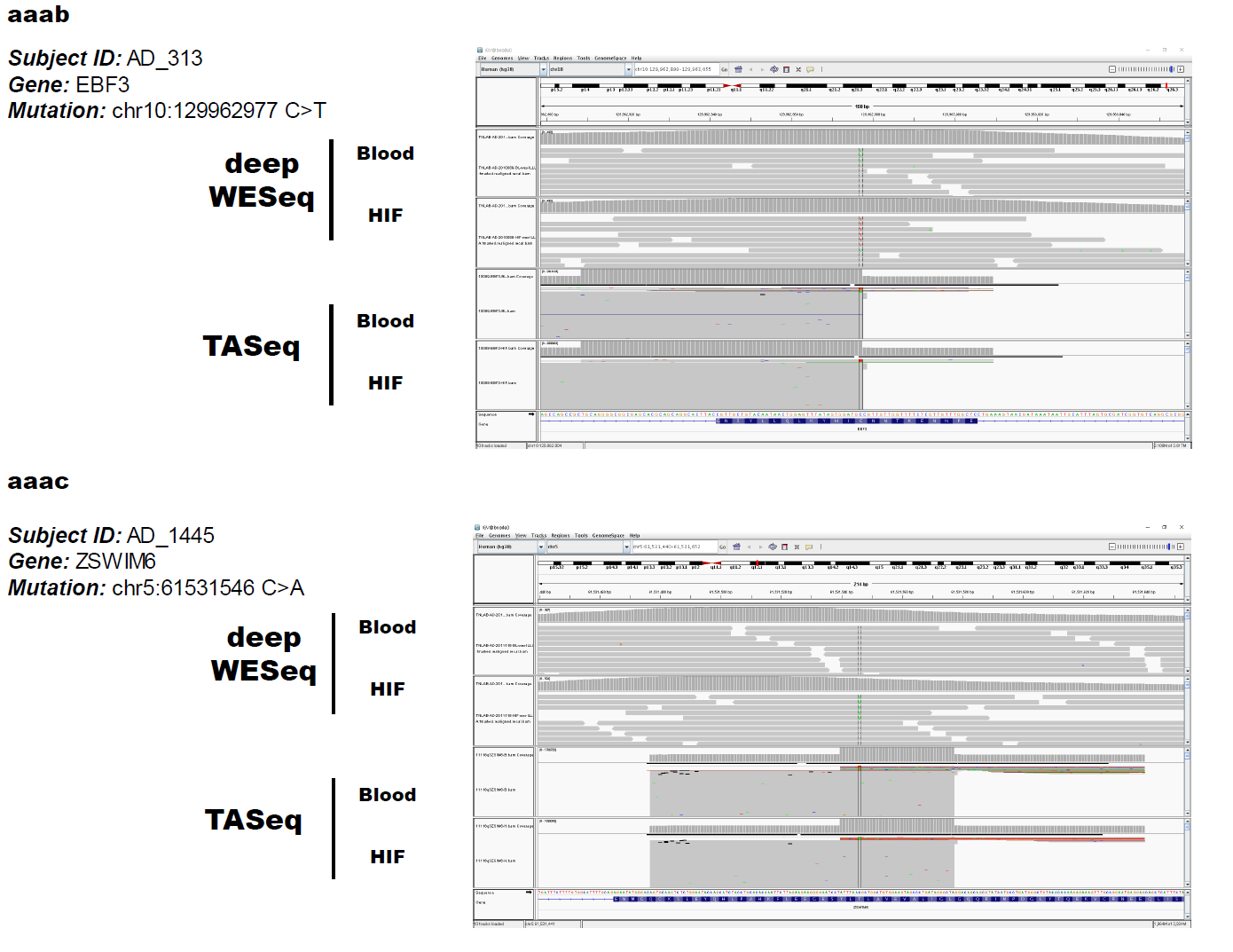


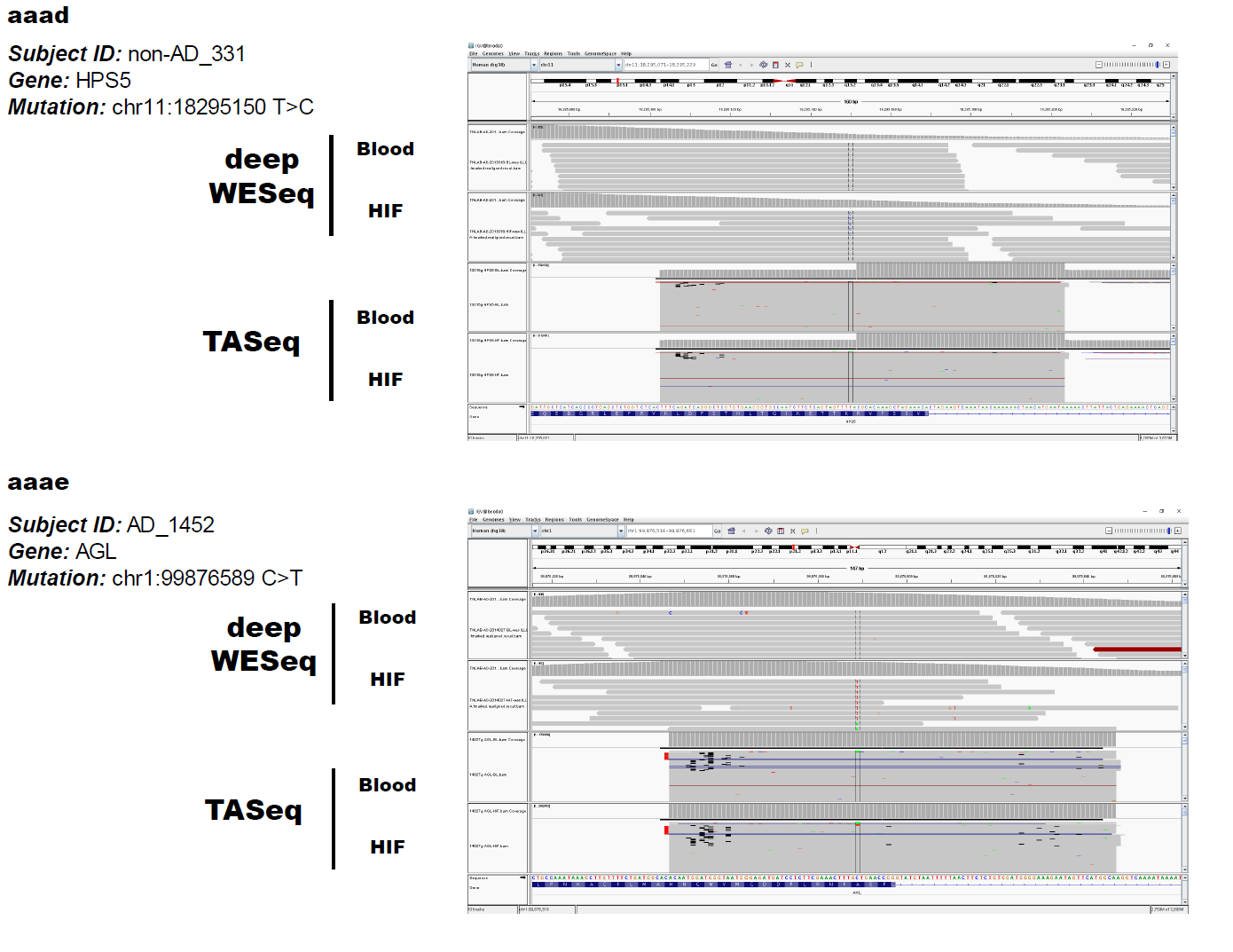


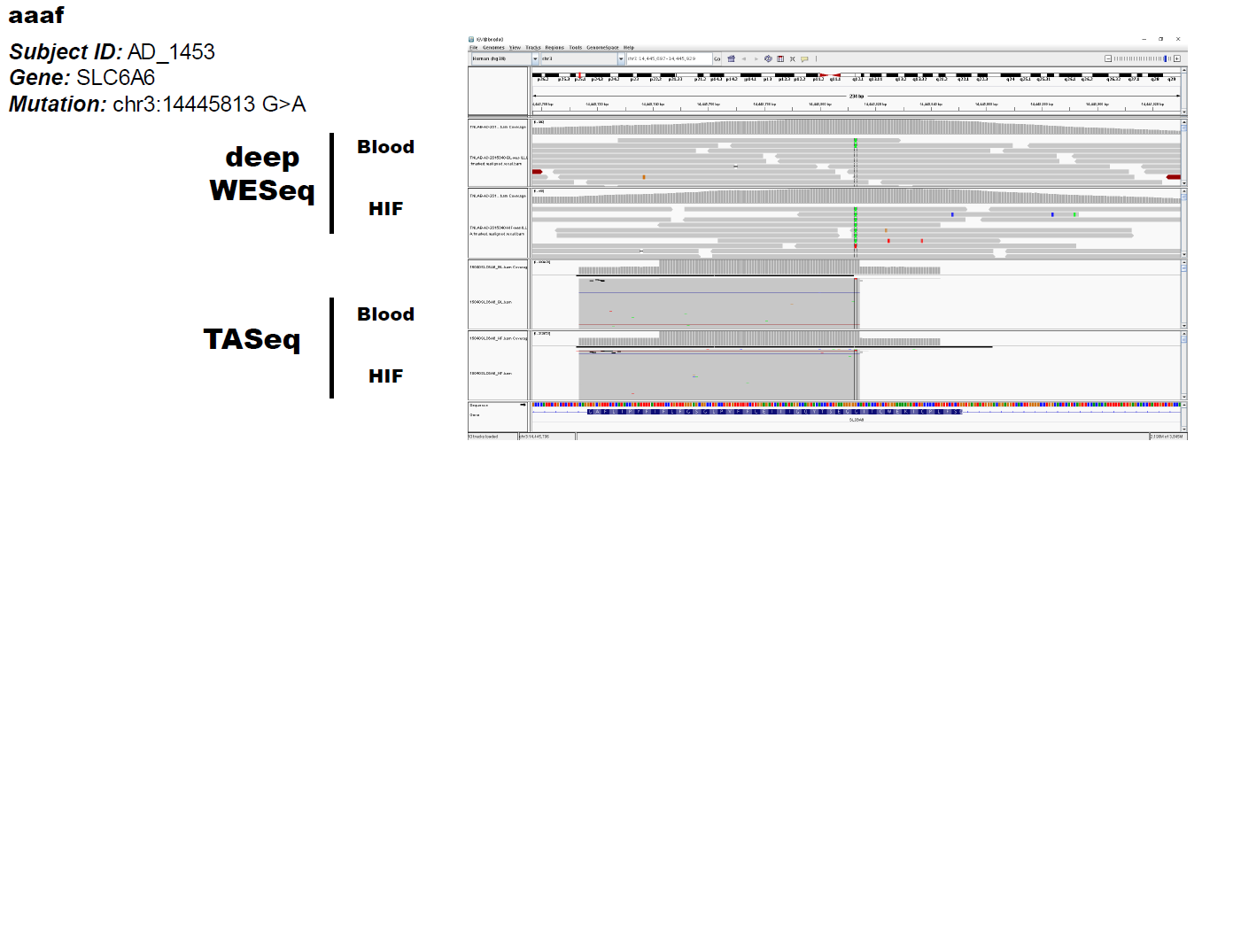


**Supplementary Figure 3:** **IGV browser images of validated brain somatic mutations.**

Deep whole exome sequencing and targeted amplicon sequencing results of 84 randomly picked brain somatic variants were aligned to human genome reference (GRCh38). Analysis-ready bam files of 67 true calls (**a~aao**) and 17 false calls (**aap~aaaf**) were snap-shot captured in parallel.


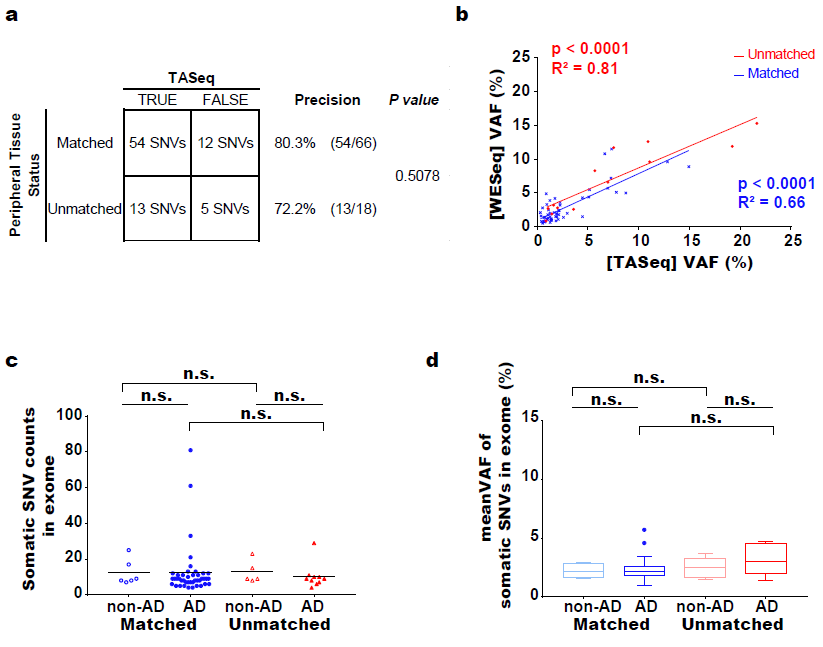


**Supplementary Figure 4: Comparison of the detection accuracy and mutation profile of unmatched and matched samples.**

**a,** Validation results of post-filtered brain somatic mutations in unmatched and matched samples using targeted amplicon sequencing. *P* value was calculated from Fisher’s exact T-test. **b,** Correlation of VAFs for true calls between deep whole exome sequencing and targeted amplicon sequencing. The line of best fit (solid line) and *P* values were calculated by linear regression. **c,** Comparison of average mutation counts of AD and non-AD individuals from unmatched and matched samples. **d,** Comparison of mean VAFs of AD and non-AD individuals from unmatched and matched samples which are shown as box plots (center line, median; box limits, upper and lower quartiles; whiskers, maximum and minimum values; points, outliers). *P* values were calculated by one-way ANOVA test, followed by post hoc multiple comparison in panels c, d. Abbreviations: n.s, not significant.


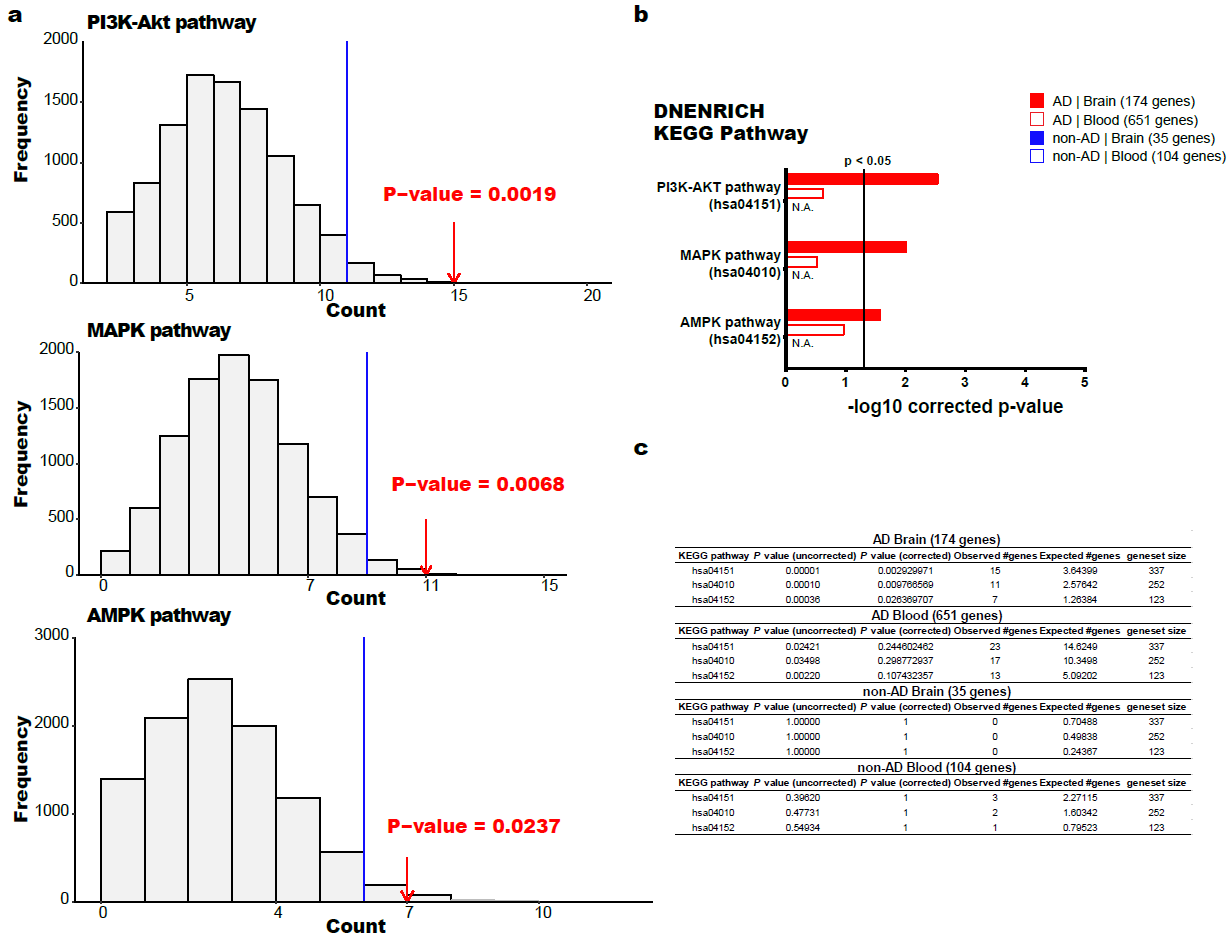


**Supplementary Figure 5: Random permutation and gene-length adjusted enrichment test of genes with putatively pathogenic somatic mutations.**

**a**, The distribution of overlapping genes was generated by randomly selecting 174 (AD Brain) genes from a reference gene list of PI3K-AKT, MAPK, and AMPK pathways. *P* values were estimated by comparing the enrichment test overlap counts (red arrow) of AD_Brain gene sets with a distribution cutoff of 5%. **b**, Gene-length adjusted enrichment test was performed with DNENRICH along with 100,000 permutations. Vertical bar represents threshold for Benjamini-Hochberg adjusted *P* value. **c**, Detailed statistical significance for gene-length adjusted enrichment of gene sets with putatively pathogenic somatic mutations. Corrected *P* values were estimated using the Benjamini-Hochberg method.

.
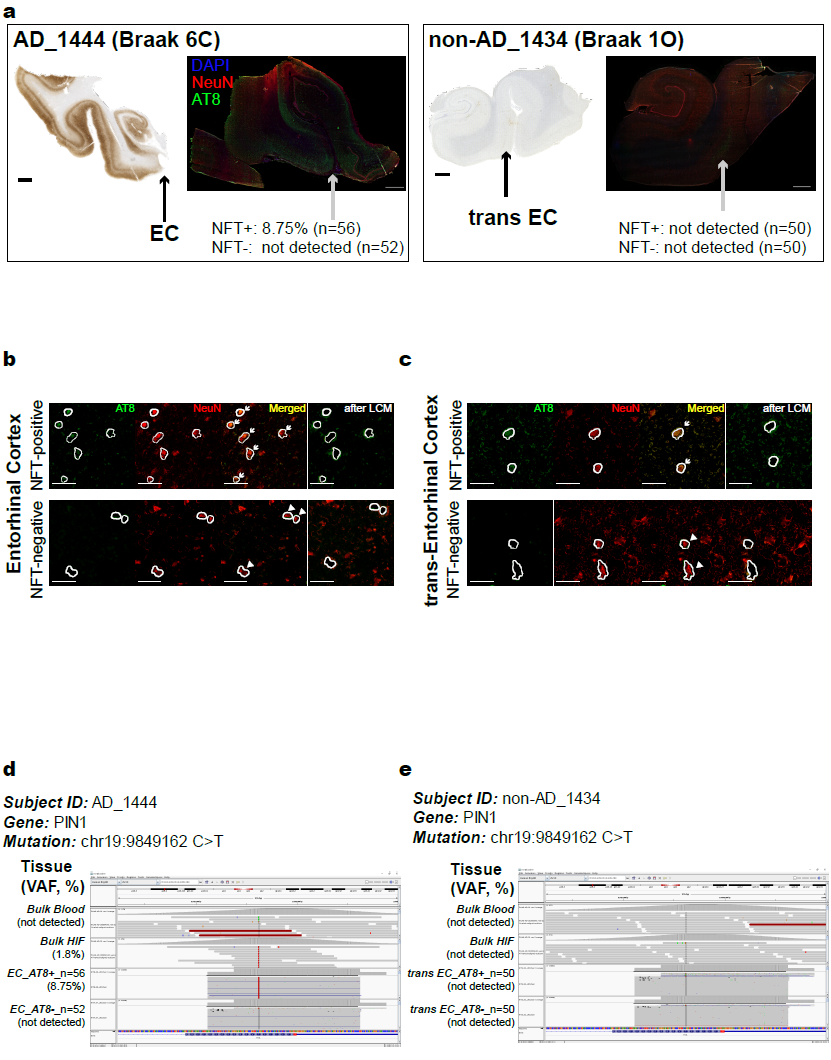


**Supplementary Figure 6: Targeted amplicon sequencing of AT8-negative/positive neurons captured with laser microdissection.**

**a,** Diaminobenzidine (DAB) and immunofluorescence staining of hyper-phosphorylated Tau in the hippocampal formations of AD_1444 (Braak Ⅵ, CERAD C) and non-AD_1434 (Braak Ⅰ, CERAD O). Scale bar, 2mm. **b, c,** Laser capture microdissection of AT8-positive (arrow) and AT8-negative (arrow head) neurons in entorhinal cortex of the AD patient with the *PIN1* c.477 C>T (p.Thr152Met) somatic mutation. In case of non-AD_1434, AT8-positive and AT8-negative neurons were isolated from trans-entorhinal cortex. Scale bar, 50μm. **d, e,** The IGV browser images of targeted amplicon sequencing for *PIN1* c.477 C>T variant. AT8-positive neurons from the entorhinal cortex of AD_1444 showed a variant allele fraction up to 8.75% in the target site. Such mutation was not detected (i.e., below background error rate) in other cells.


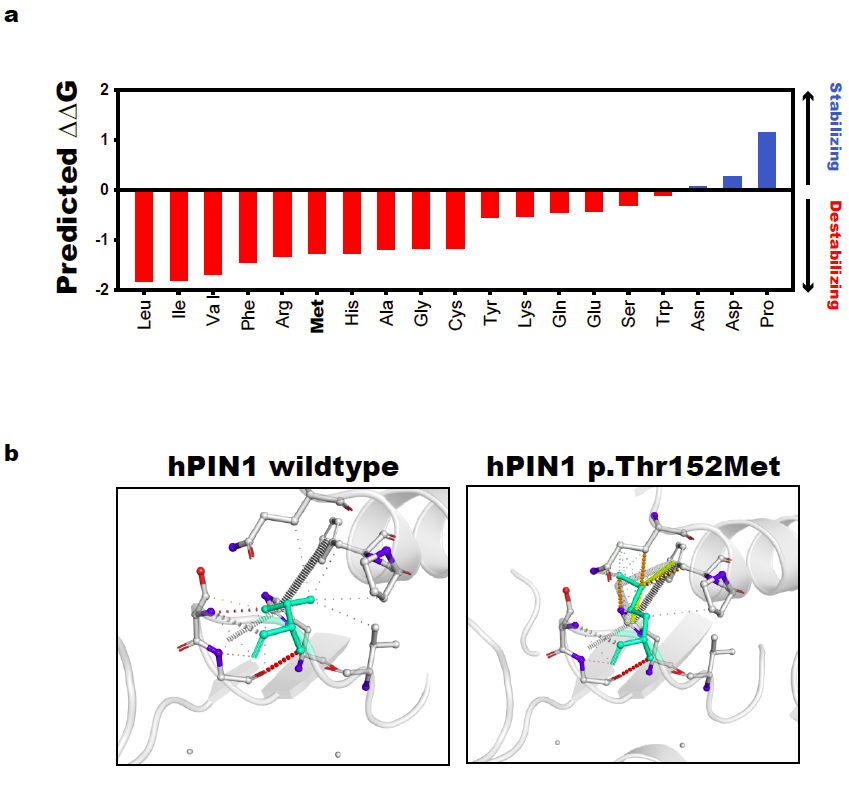


**Supplementary Figure 7: Protein structure-based stability prediction of wild-type and mutant PIN1 (p.Thr152Met).**

**a,** The predicted changes in Gibbs free energy(△△G) for 19 different possible amino acid changes in the Thr152 position of human PIN1. The stability prediction with the SDM2 algorithm showed that changing the Threonine to 16 different amino acids are destabilizing (△△G<0), whilst p.T152M was the sixth highest destabilizing mutation. **b,** Visualization of PIN1 protein with Thr152 (wild-type, left) and Met152 (mutant, right) and its surrounding protein environment. Increased solvent accessibility (yellow) and changes in hydrogen bonding of side chains (orange) can reduce the stability of mutant PIN1 protein.


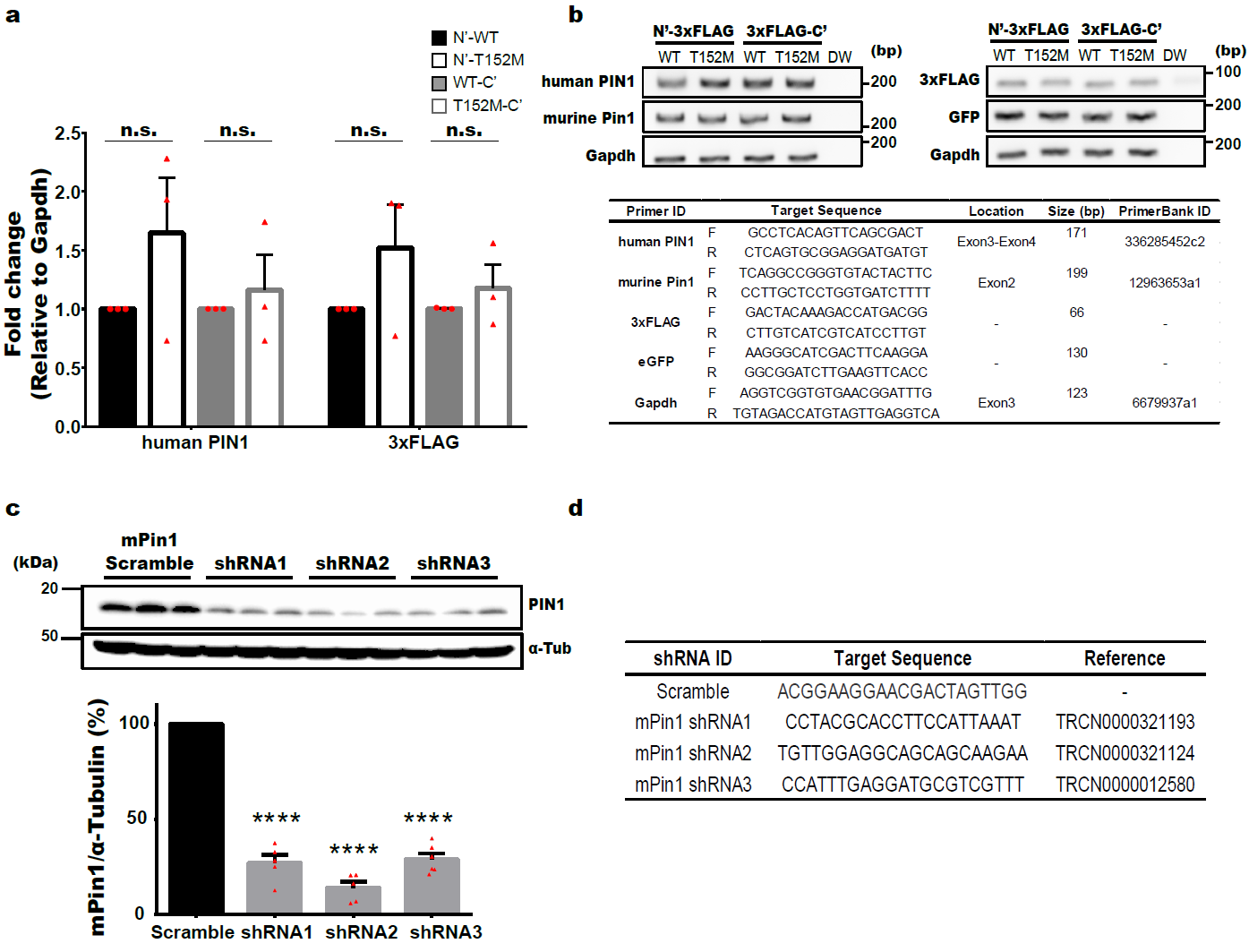


**Supplementary Figure 8: RT-qPCR analysis of human PIN1 constructs and knock-down efficiencies of murine Pin1 shRNAs.**

**a,** Fold-change in mRNA by real-time PCR. Gene expression profiles of human PIN1 and 3xFLAG relative to Gapdh in N2a cells transfected with either wild-type or mutant human PIN1 constructs. Data were measured in triplicate and represent the mean ± SEM; n=3 in each group. Statistical assessments were made using unpaired T-test. **b,** Agarose gel electrophoresis image of RT-qPCR products and list of qPCR primer sequences used in the analysis. **c,** Western blot analysis demonstrates the efficiency of murine Pin1 knockdown in N2a cells. Alpha tubulin was used as an internal control. While all of shRNAs could be used for mPin1 knockdown, the efficacy of shRNA3 was the closest to 50%. Data represent mean ± SEM; n=5-6 in each group. **d,** List of the shRNA target sequences and reference TRCN IDs used in this study against murine Pin1. *P* values were generated by one-way ANOVA test, followed by post hoc multiple comparison. Abbreviation: n.s., not significant; ****, *p*<0.0001.


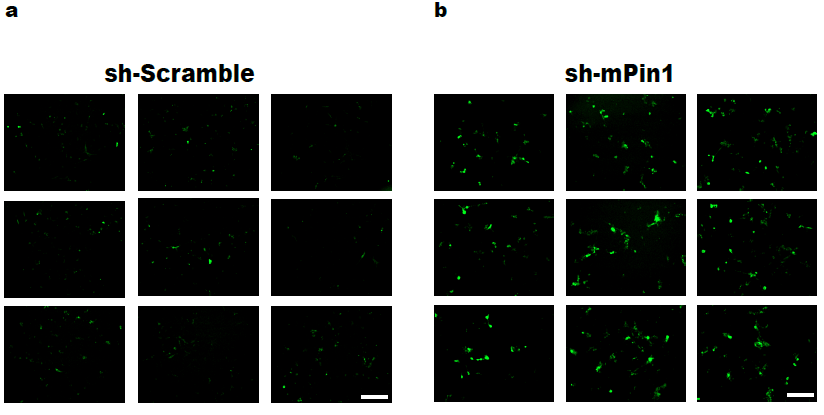


**Supplementary Figure 9: Basal fluorescence intensities of Tau-BiFC cell lines.**

Mouse hippocampal neuronal cells (HT22) were co-transfected with Tau-BiFC construct and either **a,** scramble or **b,** murine Pin1 targeting shRNA. Tau-BiFC signals were captured using an EVOS auto imaging system. Scale bar, 250μm.


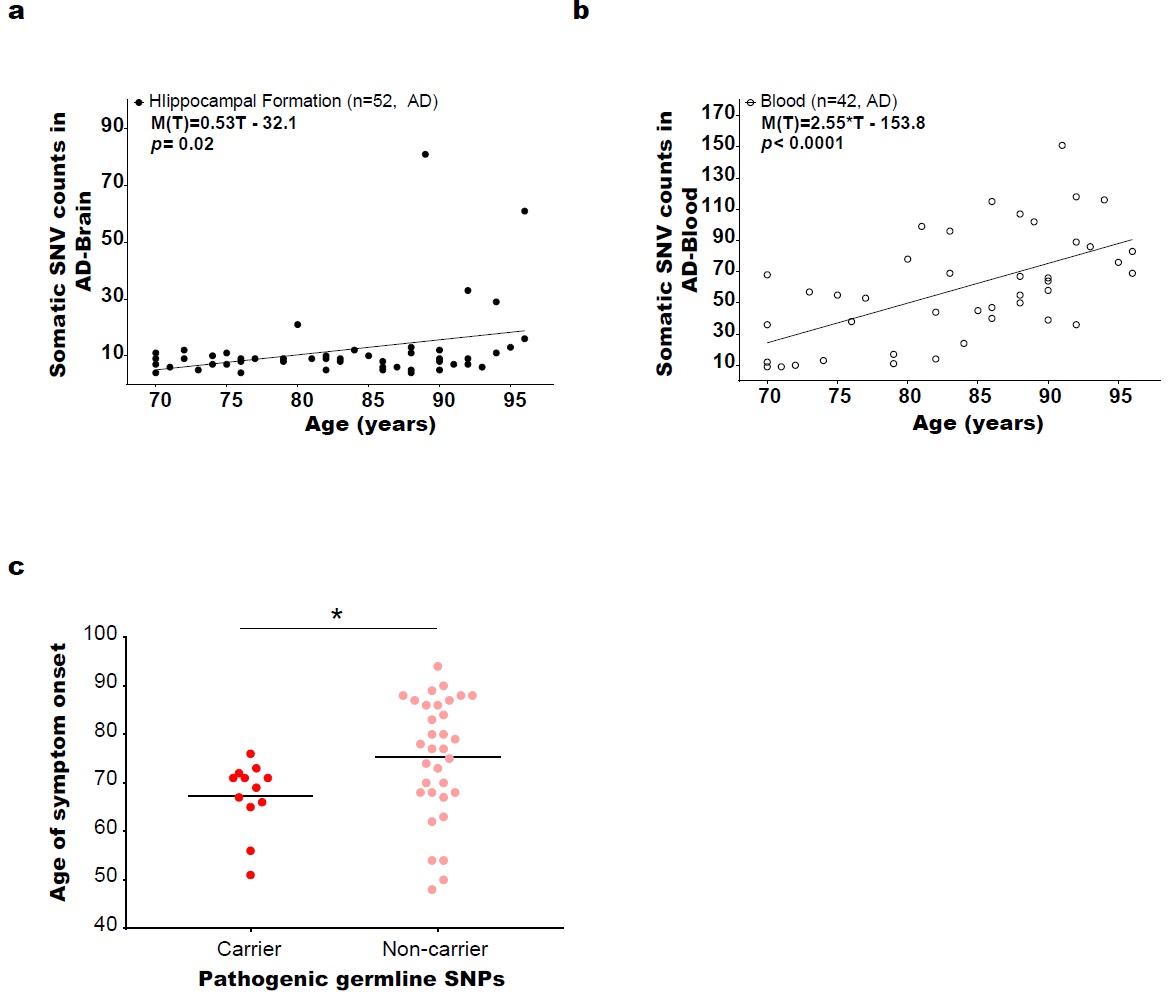


**Supplementary Figure 10: Somatic mutation accumulation speed and clinical onset timing of Alzheimer’s disease.**

**a,b,** The numbers of somatic mutations increase with aging in both brain and blood tissues from AD subjects. The brain and blood somatic mutations accumulate at rates of 0.53 SNVs per year per exome and 2.55 SNVs per year per exome. The line of best fit (solid line) and *P* values were calculated by linear regression. **c,** Mean onset time for AD (e.g., AD-onset information was available for 45/52 AD patients) depending on carrying pathogenic germline mutations. The lines represent mean of onset timing. *P* values was generated by unpaired T-tests accompanied with Welch’s correction. Abbreviation: *, *p*<0.05.


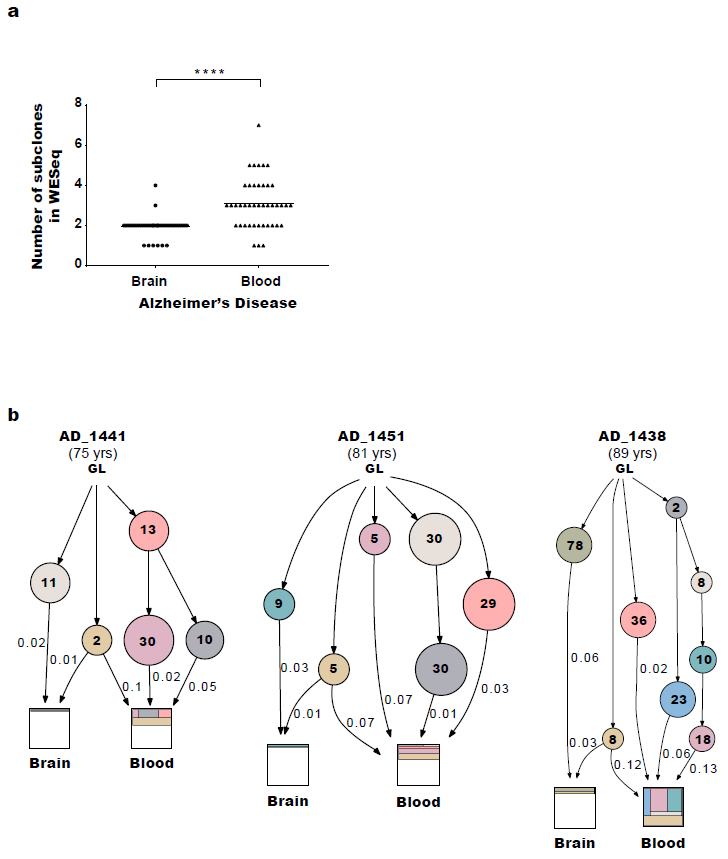


**Supplementary Figure 11: Comparison of sub-clones and inferred clonal lineage trees in brain and blood samples of Alzheimer’s disease.**

**a,** The number of sub-clones, obtained from the LICHeE algorithm in brain and blood samples of Alzheimer’s disease. **b,** Examples of inferred clonal lineage trees in brain and blood tissues from three different AD patients. Nodes represent clusters of variants based on variant allele frequency (VAF) and the edges represent pairwise ancestry relationships of sub-clones. The mean VAF of each sub-clone is denoted on the edges. Paired T-test was used to determine the significance of each experiment. Abbreviations: GL, germline; ****, *p*<0.0001.


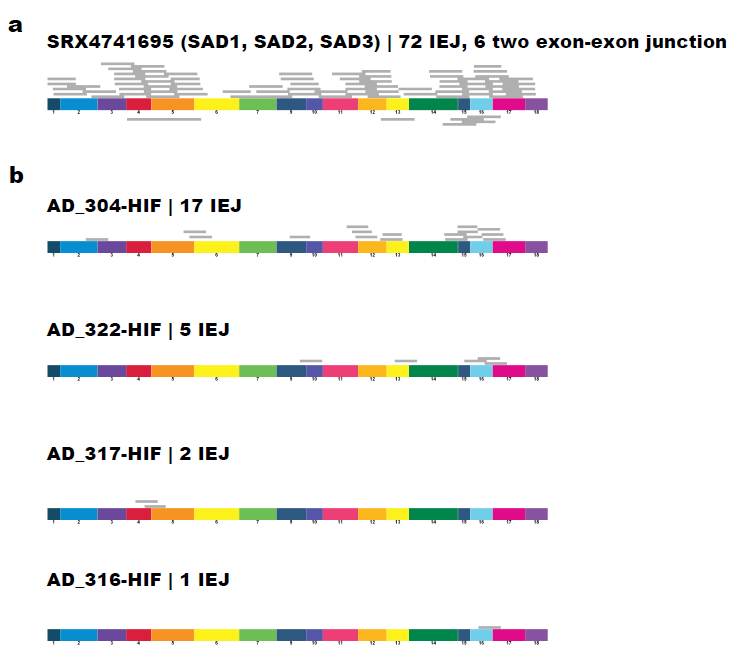


**Supplementary Figure 12:** ***APP* gencDNA identified from Lee et. al (Nature 2018) and our cohort**

**a,** Schematic of *APP* cDNA and genomic exon-exon junctions identified by Lee et. al.^1^ Analyses of reported sequencing data from three individuals with sporadic AD (e.g., 40,000 neuronal nuclei) identified 78 gencDNA including 72 intra exon-exon junction and six two-exon-exon junctions. **b,** Four different AD brain exomes in our cohort show 1-17 intra exon-exon junctions.

**Supplementary References**

1. Lee M-H*, et al.* Somatic APP gene recombination in Alzheimer’s disease and normal neurons. *Nature* **563**, 639-645 (2018).
