# Supplementary material for: Brain somatic mutations observed in Alzheimer’s disease associated with aging and dysregulation of tau phosphorylation
Source: Nat Commun. 2019 Jul 12;10:3090. doi: 10.1038/s41467-019-11000-7 (PMC6626023; doi:10.1038/s41467-019-11000-7)
Supplement: Supplementary file 3 — Description of Additional Supplementary Files [file 41467_2019_11000_MOESM3_ESM.pdf]

Description of Additional Supplementary Files

File Name: Supplementary Data 1
Description: Detailed clinico-pathological and sequencing result descriptions of AD and non-AD subjects

File Name: Supplementary Data 2
Description: Detailed information on deep whole exome sequencing results of non-SCZ and SCZ subjects

File Name: Supplementary Data 3
Description: Targeted amplicon sequencing results for determining the cut-off value of EBscore

File Name: Supplementary Data 4
Description: Targeted amplicon sequencing results and related primer sequences for randomly selected brain somatic mutations

File Name: Supplementary Data 5
Description: List of post-filtered brain and blood somatic single nucleotide variations in AD and non-AD

File Name: Supplementary Data 6
Description: PCAWG signatures adjusted to human whole-exome trinucleotide frequencies

File Name: Supplementary Data 7
Description: Detailed summary report of mutation signature analyses of AD brain and AD blood from Mutalisk tool

File Name: Supplementary Data 8
Description: List of putatively pathogenic brain and blood somatic single nucleotide variations in AD and non-AD

File Name: Supplementary Data 9
Description: List of brain somatic mutations in AD with CADD Phred score and the number of evidence sentences from the DigSee engine

File Name: Supplementary Data 10
Description: Targeted amplicon sequencing results of a PIN1 (c.477 C>T) mutation from bulk, sub-region, and AT8-positive/negative entorhinal cortical neurons of the hippocampal formation

File Name: Supplementary Data 11
Description: List of pathogenic germline risk factors and Tau-pathology affecting putatively pathogenic somatic mutations in AD and non-AD

File Name: Supplementary Data 12
Description: Variant allelic frequencies, mutation context, and predicted $\Delta\Delta G$ of AD brain somatic mutations enriched in PI3K-AKT, MAPK, and AMPK pathways

File Name: Supplementary Data 13
Description: List of read counts of 22 variants from Wei et. al[1] (Genetics in Medicine, 2018) and the nine variants from Nicolas et. al[2] (Alzheimer's & Dementia, 2018) in our cohorts

File Name: Supplementary Movie 1
Description: Demonstration of laser capture microdissection (LCM) to isolate Nissl-stained sections of the dentate gyrus
