# Supplementary material for: Brain somatic mutations observed in Alzheimer’s disease associated with aging and dysregulation of tau phosphorylation
Source: Nat Commun. 2019 Jul 12;10:3090. doi: 10.1038/s41467-019-11000-7 (PMC6626023; doi:10.1038/s41467-019-11000-7)
Supplement: Supplementary file 18 — Reporting Summary [file 41467_2019_11000_MOESM18_ESM.pdf]

nature research

Corresponding author(s): Jeong Ho Lee, Seok Jong Yu

Last updated by author(s): May 30, 2019

Reporting Summary

Nature Research wishes to improve the reproducibility of the work that we publish. This form provides structure for consistency and transparency in reporting. For further information on Nature Research policies, see Authors & Referees and the Editorial Policy Checklist.

Statistics

For all statistical analyses, confirm that the following items are present in the figure legend, table legend, main text, or Methods section.

n/a	Confirmed	
☐	☒	The exact sample size (*n*) for each experimental group/condition, given as a discrete number and unit of measurement
☐	☒	A statement on whether measurements were taken from distinct samples or whether the same sample was measured repeatedly
☐	☒	The statistical test(s) used AND whether they are one- or two-sided<br>*Only common tests should be described solely by name; describe more complex techniques in the Methods section.*
☐	☒	A description of all covariates tested
☐	☒	A description of any assumptions or corrections, such as tests of normality and adjustment for multiple comparisons
☐	☒	A full description of the statistical parameters including central tendency (e.g. means) or other basic estimates (e.g. regression coefficient) AND variation (e.g. standard deviation) or associated estimates of uncertainty (e.g. confidence intervals)
☐	☒	For null hypothesis testing, the test statistic (e.g. *F*, *t*, *r*) with confidence intervals, effect sizes, degrees of freedom and *P* value noted<br>*Give P values as exact values whenever suitable.*
☒	☐	For Bayesian analysis, information on the choice of priors and Markov chain Monte Carlo settings
☒	☐	For hierarchical and complex designs, identification of the appropriate level for tests and full reporting of outcomes
☐	☒	Estimates of effect sizes (e.g. Cohen's *d*, Pearson's *r*), indicating how they were calculated

Our web collection on statistics for biologists contains articles on many of the points above.

Software and code

Policy information about availability of computer code

Data collection	- Combined Annotation Dependent Depletion (CADD) GRCh38-v1.4, genome Aggregation Database (gnomAD) r.2.0.2: freely available for academic purpose. Method paragraph 4, 6<br>- Exome-adjusted PCAWG reference SBS signatures: provided by Dr. Ludmil Alexandrov (UC San Diego). Supplementary Table 6<br>- Reference genes associated Kyoto Encyclopedia of Genes and Genome (KEGG) pathways v.2016 (http://amp.pharm.mssm.edu/Enrichr/geneSetLibrary?mode=text&libraryName=KEGG_2016): freely available for academic purpose. Methods; p 28, paragraph 1, 2<br>- Pathogenic mutation information of genes associated with Alzheimer's disease (AlzGene, https://www.alzforum.org/mutations): freely available for academic purpose. Methods; p 32, paragraph 1<br>- Targeted gene panel sequencing data (SRX4741695) for validating APP gencDNA reported in Lee et. al [PMID 30464338]: freely available in (https://www.ncbi.nlm.nih.gov/sra/?term=SRX4741695). Supplementary Fig. 12; Methods, p 32, paragraph 3
Data analysis	- R packages (pROC-v.1.12.1, ggplot2-2.2.1, exonjunction): freely available for academic purpose: to display and perform statistical analyses on the data<br>- Graphpad Prism v.7: from GraphPad Software, USA: to display and perform statistical analyses on the data<br>- PALM Robo software v4.6: from Zeiss, Germany. Methods; p 23, paragraph 2; p 27, paragraph 1<br>- ImageJ v.1.50: freely available for academic purpose. Methods; p 24, paragraph 1; p 31, paragraph 3<br>- Bioanalyzer 2100 Expert software B.02.08: from Agilent, USA. Methods; p 23, paragraph 2; p 26, paragraph 1<br>- FastQC v.0.11.3, BWA-mem v.0.7.15, Picard v.2.2.1, SAMtools v.1.3.1, GATK best v.3.5.0, dbSNP v.147, COSMIC v.77, GRCh38/hg38 GCA_000001405.15, Vecuum v1.0.1, MuTect v1.1.7: freely available for academic purpose. Methods; p 24, paragraph 2-3<br>- Empirical Bayesian Mutation Filtering (EBFilter) v.0.2.1: freely available for academic purpose. Methods; p 25, paragraph 2<br>- Variant Effect Predictor (VEP) v.94: freely available for academic purpose. Methods; p 25, paragraph 1<br>- Integrative Genomic Viewer (IGViewer) v.2.3: freely available for academic purpose. Methods; p 39, paragraph 2; p 41, paragraph 1<br>- bam-readcount v.0.8.0: freely available for academic purpose. Methods; p 25, paragraph 1; p 26, paragraph 1<br>- RePlow v.1.0.0: freely available for academic purpose. to estimate significance of targeted amplicon sequencing data by comparing to background error rates of the PCR-based, amplicon-based platforms. Methods; p 26, paragraph 1<br>- Primer3 v.0.4.0: freely available for academic purpose. Methods; p 26, paragraph 1; p 27, paragraph 1;

- Mutation Analysis Toolkit (Mutalisk): freely available for academic purpose. Methods; p 27, paragraph 2
- Disease Gene Search Engine with Evidence Sentences (DigSee) v.2.01 : freely available for academic purpose. Methods; p 27, paragraph 3
- Site Directed Mutator (SDM) v.2: freely available for academic purpose. Methods; p 28, paragraph 1
- Enrichr: freely available for academic purpose. Methods; p 28, paragraph 2
- DNENRICH: freely available for academic purpose. Methods; p 28, paragraph 2
- CFX Manager software v.3.1: from Bio-Rad, USA. Methods; p 30, paragraph 1
- EVOS FL Auto Cell Imaging System Software v1.7 from Thermo Fisher, USA : Methods; p 31, paragraph 3
- LICHeE: freely available for academic purpose. Methods; p 32, paragraph 2

For manuscripts utilizing custom algorithms or software that are central to the research but not yet described in published literature, software must be made available to editors/reviewers. We strongly encourage code deposition in a community repository (e.g. GitHub). See the Nature Research guidelines for submitting code & software for further information.

Data

Policy information about availability of data

All manuscripts must include a data availability statement. This statement should provide the following information, where applicable:

- Accession codes, unique identifiers, or web links for publicly available datasets
- A list of figures that have associated raw data
- A description of any restrictions on data availability

All datasets generated and/or analyzed during the current study are presented in this article or the accompanying Source Data or Supplementary Information files will be available from the corresponding authors upon request. 111 Deep whole-exome sequencing data produced in the current study have been deposited in the NCBI Sequence Read Archive (SRA) with accession number PRJNA532465. Targeted amplicon sequencing data for randomly chosen validation process and confirmation of putative pathogenic variant of PIN1 (c.477 C>T) are deposited in the SRA with accession numbers PRJNA532989 and PRJNA532992, respectively.

Field-specific reporting

Please select the one below that is the best fit for your research. If you are not sure, read the appropriate sections before making your selection.

☒ Life sciences ☐ Behavioural & social sciences ☐ Ecological, evolutionary & environmental sciences

For a reference copy of the document with all sections, see nature.com/documents/nr-reporting-summary-flat.pdf

Life sciences study design

All studies must disclose on these points even when the disclosure is negative.

Sample size

Human sample:
To our knowledge, our cohort consisting of 52 Alzheimer's disease patients and 11 non-demented control with matched samples, which include hippocampal formation (HIF) and blood, is the largest one for the deep whole exome sequencing based genomic assessment of aged individuals. As independently acquiring such freshly frozen brain tissue and peripheral fluid samples was limited, we tried to get as much as samples we could afford. Thereby, no statistical methods were used for pre-determining sample sizes.

in vitro cell line sample:
All experiments with cell lines were conducted with multiple biological replicates and based on previous experiences with specific experimental set-up.

Animal sample:
No animal studies reported in the study.

Data exclusions

No tissues or in vitro cell line samples were excluded from analyses.

Replication

All attempts at replication were successful.

Human sample:
Deep whole-exome sequencing of human was validated with independent high-throughput targeted amplicon sequencing (See Method).

in vitro cell line sample:
Reported results were tested and confirmed in at least two independent cell lines in addition to repeating the experiments between 3 to 9 times.

Animal sample:
No animal studies reported in the study.

Randomization

Human sample:
Samples were organized into two groups based on clinico-pathological indication of Alzheimer's disease or non-demented controls.

in vitro cell line sample:
Samples were organized into two groups based on overexpressing wildtype or mutant human PIN1 and whether they were treated with murine Pin1 shRNA or scramble shRNA

Animal sample:
No animal studies reported in the study.

Blinding

Human genetics:
Investigators were not blinded to group allocation. Importantly, the bioinformatic analyses were performed using the same script.

in vitro cell line sample:
Investigators were blinded to perform quantification of BiFC signal (Method paragraph 15, Fig. 3f and Supplementary Fig. 7).

Animal sample:
No animal studies reported in the study.

Reporting for specific materials, systems and methods

We require information from authors about some types of materials, experimental systems and methods used in many studies. Here, indicate whether each material, system or method listed is relevant to your study. If you are not sure if a list item applies to your research, read the appropriate section before selecting a response.

Materials & experimental systems

n/a	Involved in the study	
☐	☒	Antibodies
☐	☒	Eukaryotic cell lines
☒	☐	Palaeontology
☒	☐	Animals and other organisms
☐	☒	Human research participants
☒	☐	Clinical data

Methods

n/a	Involved in the study	
☒	☐	ChIP-seq
☒	☐	Flow cytometry
☒	☐	MRI-based neuroimaging

Antibodies

Antibodies used

Commercial antibodies were used in the study.
Following primary antibodies were used,

PHF-Tau (AT8) (1:500, MN1020, Thermo)
NeuN (1:500, ab104225, Abcam)
alpha Tubulin (1:1000; 3873, Cell Signaling)
DYKDDDDK Tag (FLAG) (1:1000, 8146, Cell Signaling)
Pin1 (1:500, 46660, Santa Cruz)
Total Tau protein (Tau5) (1:1000, AHB0042, Thermo Fisher)
Phospho-Tau, Thr231 (AT180) (1:1000, MN1040, Thermo Fisher)
beta Actin (1:2000, 3700, Cell Signaling)

Following secondary antibodies were used,

Goat Anti-Mouse IgG (H+L), Alexa Flour 488 (1:500, A11001, Thermo)
Goat Anti-Rabbit IgG (H+L), Alexa Flour 594 (1:500, A11012, Thermo)
Anti-rabbit IgG, HRP-linked (1:10,000, 7074, Cell Signaling)
Anti-mouse IgG, HRP-linked (1:10,000, 7076, Cell Signaling)

Validation

Primary antibodies were validated by westernblot and immunoflouresence in house and also validated by the manufacturers.

Eukaryotic cell lines

Policy information about cell lines

Cell line source(s)

- Immortalized mouse neuronal cell line (Neuro-2a, ATCC® CCL-131™, https://www.atcc.org/products/all/CCL-131.aspx)
- Immortalized mouse hippocampal neuronal cell line (HT22, Davis, J.B. & Maher, P. Protein kinase C activation inhibits glutamate-induced cytotoxicity in a neuronal cell line. Brain Research 652, 169-173 (1994))

Authentication

- We purchased Neuro-2a directly from ATCC. The supplier, ATCC, authenticate the cell line.
- The mouse hippocampal neuronal cell line HT22 was a gifted from Dr. David Schubert (Salk Institute) to I. M-J.

Mycoplasma contamination

We tested weekly for mycoplasma contamination using e-Myco plus Mycoplasma PCR Detection kit (Intron, Korea). All cell lines were mycoplasma-negative.

Commonly misidentified lines
(See ICLAC register)

No commonly misidentified cell lines were used

Human research participants

Policy information about studies involving human research participants

Population characteristics	- The number of patients with Alzheimer's disease were 52. Mean age was 83.06 ± 7.96 (range, 70-96). Mean onset-age was 73.18 ± 11.67 (range, 48-94). The numbers of male and female were 16 and 36, respectively.<br>- The number of age-matched non-demented controls were 11. Mean age was 74.09 ± 9.43 (range, 57-89). The numbers of male and female were 7 and 4, respectively.<br>- The number of non-demented, Schizophrenia patients were 11. Mean age was 46.27 ± 6.45 (range, 37-59). Mean onset-age was 19.91 ± 4.37 (range, 14-30). The numbers of male and female were 7 and 4, respectively.<br>- The number of non-demented, non-Schizophrenia healthy controls were 21. Mean age was 45.95 ± 7.92 (range, 31-63). The numbers of male and female were 19 and 2, respectively.
Recruitment	Autopsized brain tissue and matched blood samples were obtained from the Netherlands Brain Bank (NBB, https://www.brainbank.nl) and the Human Brain and Spinal Fluid Resource Center (HBSFRC, http://brainbank.ucla.edu). Genomic DNA isolated from brain and liver tissues of Schizophrenia and non-Schizophrenia healthy controls were obtained from the Stanley Medical Research Institute (SMRI, http://www.stanleyresearch.org/). The NBB, HBSFRC, and SMRI obtained permission from the donors for brain autopsy and use of tissue, blood, and clinical information for research purposes. The study was performed with informed consent according to protocols approved by Institutional Review Boards of KAIST, as well as the Committee on Human research.
Ethics oversight	Korea Advanced Institute of Science and Technology (IRB #: KH2014-36)

Note that full information on the approval of the study protocol must also be provided in the manuscript.
